# B cells in the pneumococcus-infected lung are heterogeneous and require CD4^+^ T cell help including CD40L to become resident memory B cells

**DOI:** 10.3389/fimmu.2024.1382638

**Published:** 2024-04-23

**Authors:** Neelou S. Etesami, Kimberly A. Barker, Anukul T. Shenoy, Carolina Lyon De Ana, Emad I. Arafa, Gabrielle N. Grifno, Adeline M. Matschulat, Michael E. Vannini, Riley M. F. Pihl, Michael P. Breen, Alicia M. Soucy, Wesley N. Goltry, Catherine T. Ha, Hanae Betsuyaku, Jeffrey L. Browning, Xaralabos Varelas, Katrina E. Traber, Matthew R. Jones, Lee J. Quinton, Paul J. Maglione, Hadi T. Nia, Anna C. Belkina, Joseph P. Mizgerd

**Affiliations:** ^1^ Pulmonary Center, Boston University Chobanian and Avedisian School of Medicine, Boston, MA, United States; ^2^ Department of Virology, Immunology, and Microbiology, Boston University Chobanian and Avedisian School of Medicine, Boston, MA, United States; ^3^ Department of Microbiology and Immunology, University of Michigan Medical School, Ann Arbor, MI, United States; ^4^ Department of Biomedical Engineering, Boston University College of Engineering, Boston, MA, United States; ^5^ Department of Biochemistry and Cell Biology, Boston University Chobanian and Avedisian School of Medicine, Boston, MA, United States; ^6^ Department of Medicine, Boston University Chobanian and Avedisian School of Medicine, Boston, MA, United States; ^7^ Division of Infectious Diseases and Immunology, University of Massachusetts Chan Medical School, Worcester, MA, United States; ^8^ Department of Pathology and Laboratory Medicine, Boston University Chobanian and Avedisian School of Medicine, Boston, MA, United States; ^9^ Flow Cytometry Core Facility, Boston University Chobanian and Avedisian School of Medicine, Boston, MA, United States

**Keywords:** mucosal immunity, lung immunology, pneumonia, adaptive immunity, B cells

## Abstract

Recovery from respiratory pneumococcal infections generates lung-localized protection against heterotypic bacteria, mediated by resident memory lymphocytes. Optimal protection in mice requires re-exposure to pneumococcus within days of initial infection. Serial surface marker phenotyping of B cell populations in a model of pneumococcal heterotypic immunity revealed that bacterial re-exposure stimulates the immediate accumulation of dynamic and heterogeneous populations of B cells in the lung, and is essential for the establishment of lung resident memory B (B_RM_) cells. The B cells in the early wave were activated, proliferating locally, and associated with both CD4^+^ T cells and CXCL13. Antagonist- and antibody-mediated interventions were implemented during this early timeframe to demonstrate that lymphocyte recirculation, CD4^+^ cells, and CD40 ligand (CD40L) signaling were all needed for lung B_RM_ cell establishment, whereas CXCL13 signaling was not. While most prominent as aggregates in the loose connective tissue of bronchovascular bundles, morphometry and live lung imaging analyses showed that lung B_RM_ cells were equally numerous as single cells dispersed throughout the alveolar septae. We propose that CD40L signaling from antigen-stimulated CD4^+^ T cells in the infected lung is critical to establishment of local B_RM_ cells, which subsequently protect the airways and parenchyma against future potential infections.

## Introduction

Lower respiratory infections are among the greatest sources of morbidity and mortality worldwide, and the threat of pandemics caused by highly transmissible respiratory infections is a significant concern (1). Given that the respiratory function of the lung concomitantly exposes the lung to airborne pathogens, allergens, and pollutants, the healthy adult lung is primed to develop a rich resident adaptive arm of immunity, providing rapid and effective anamnestic responses against lung pathogens (2, 3). This is exemplified by the human nasopharyngeal pathobiont, *Streptococcus pneumoniae* (Sp), which is the most common bacterial cause of community-acquired pneumonia (CAP). Although colonization is ubiquitous and asymptomatic among healthy adults, young children with underdeveloped immune systems or adults with eroded barrier immunity secondary to aging, immunocompromising conditions, or chronic lung disease are significantly more susceptible to contracting pneumonia (4).

Pneumococcal vaccines have curtailed the incidence and mortality rates of CAP among susceptible populations (5). However, the systemic protection elicited by pneumococcal vaccines is limited to the recognition of the capsular polysaccharides of serotypes included in the vaccine formulae. Since non-vaccine serotypes also cause disease, there would be a benefit to serotype-independent immunization strategies that can recapitulate the natural heterotypic immunity seen in healthy adults (6, 7). Further, despite containing serotype 3 capsular polysaccharide antigen in their formulae, current vaccines do not effectively prevent infection by Sp3, which often causes severe clinical manifestations in humans (8).

T resident memory (T_RM_) cells are critical orchestrators of rapid mucosal memory recall responses, including local serotype-independent immunity to pneumococcus (9–12). Increased appreciation for the extent of host mucosal and systemic immune compartmentalization over time led to recent studies demonstrating that B_RM_ cells can also be established after local infection and contribute to the clearance of respiratory viruses and bacteria (13–15). This population is poised to rapidly differentiate into IgM^+^ and class-switched (CSW) antibody-secreting cells positioned to directly secrete local antibodies upon antigen reexposure (14, 16). Further, lung B cell transcriptomes, immunoglobulin specificities, and clonal repertoires are specialized and demonstrably different from those of B cells in circulation and secondary lymphoid organs, and can confer superior neutralizing activity against respiratory pathogens (17, 18). These properties of B_RM_ cells highlight their potential to contribute to mucosal vaccine responses; leveraging their activity will require knowledge of the optimal conditions for triggering and supporting B resident memory in the lung.

We previously showed that murine lung PD-L2^+^ B_RM_ cells formed after respiratory exposures to self-limiting *S. pneumoniae* serotype 19F (Sp19F) contribute to heterotypic protection against the otherwise lethal *S. pneumoniae* serotype 3 (Sp3) likely via the secretion of lung-localized Sp-reactive antibodies (14). Our findings dovetailed with a contemporary study revealing the presence of heterotypic influenza A virus (IAV) antigen-specific lung B_RM_ cells established in IAV-experienced mice (13). While the humoral aspect of their protective function and requirement for local exposure to antigen has been recognized, there are many knowledge gaps about lung B cells that remain. Parameters such as their dynamics, locations, subset heterogeneity, and molecular requirements for induction and maintenance are still not well known and have predominantly been studied in mouse IAV models (19). Lung immune and architectural elements are altered in distinct ways depending on the triggering pathogen (20–22). It was initially proposed that B_RM_ cells require the presence of mature tertiary lymphoid structures called induced bronchus-associated lymphoid tissues (iBALT), which are robustly generated in mouse IAV models and support a sizeable lasting population of germinal center (GC) B cells (23, 24). However, it was later determined that lung memory B cells (MBCs) can also be harbored in immature or poorly organized perivascular infiltrates after Sp infections or sterile particulate injury (14, 25), and in lungs with iBALT, B_RM_ cells can also be spread discretely across alveolar regions (16). The lack of mature iBALT formed in the Sp immune infection model represents a distinct context for MBC establishment and recall in tissues, mirroring responses of healthy human lungs in which iBALT formation is limited (12, 14). Human lungs are certainly antigen-experienced and do contain MBCs bearing lymphocyte residency markers, further emphasizing the importance of studying B_RM_ cells in respiratory exposure models that shape the lung immune response in different ways (14, 26).

Using the model of local serotype-independent immunity generated by self-limiting exposures to Sp (12), we interrogated lung extravascular (EV) B cell populations over the course of their development, specifically focusing on their phenotypes and locations as well determining when and which signals were needed for lung B_RM_ cell establishment.

## Results

### Diverse and dynamic lung B cell populations precede the formation of B_RM_ cells

**Figure 1:**
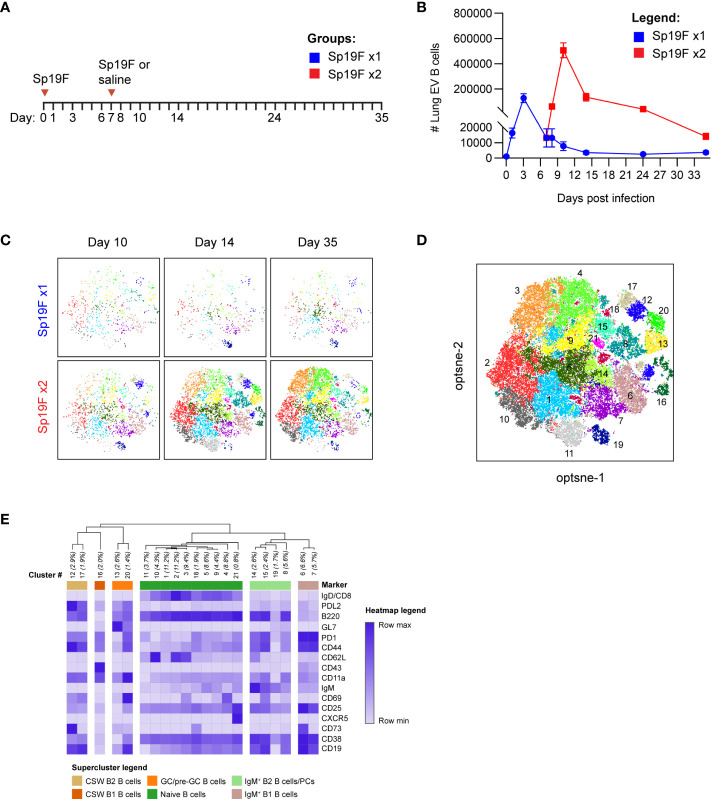
Heterogeneous lung EV B cell populations generated after Sp19F infections. **(A)** Timeline to study lymphocyte kinetics after 1 or 2 Sp19F infections. Adult B6 mice received intratracheal (i.t.) instillations of Sp19F or saline into the left lung on day 0 and day 7 and were rested for 4 weeks to generate immunity. **(B)** Average numbers of lung EV B cells (i.v.CD45^-^CD45^+^CD3^-^CD4^-^CD19^+^ B cells) at each collection timepoint, n=6 per timepoint, 2 biological replicates. **(C)** Individual op-tSNE plots with overlaid PhenoGraph clusters depicting live EV B cells after 1 or 2 infections with Sp19F on day 10, day 14, and day 35; n=6 per group. **(D)** Merged opt-SNE plot from each of the projections in panel **(C)** with overlaid and labeled PhenoGraph clusters. **(E)** Heatmap of average MFI per marker normalized to minimal MFI and maximal MFI generated with Morpheus (28). Hierarchical clustering and dendrogram construction were performed using Pearson’s correlation distances.

Self-limiting Sp19F infections, resulting from two Sp19F instillations into the left lung lobe spaced 1 week apart, confer left lung-localized heterotypic immunity against an otherwise fatal Sp3 infection occurring a month or more later (12). We have previously demonstrated roles for Th17^+^ T_RM_ and B_RM_ cells in mediating heterotypic protection, with B_RM_ cells remaining in the ipsilaterally-experienced lobe for up to 6 months after two Sp19F infections (12, 14, 27). Mice that receive only 1 respiratory Sp19F exposure (denoted as Sp19F x1), saline, or contralateral lung Sp3 challenge are not protected (12). We hypothesized that the second Sp19F infection (denoted as Sp19F x2) is necessary for the recruitment and phenotypic diversity of B cells in the lung, including their progression to resident B_RM_ cells. To test this, we assembled a 22-color lymphocyte surface marker panel for full spectrum flow cytometric analysis of lungs collected at 8 different timepoints from day 0 to day 35, including groups that received either one or two Sp19F infections (**Figure 1A**) (29). Intravascular (IV) administration of a fluorescently-tagged CD45.2 antibody shortly before euthanasia of each animal allowed for distinction between extravascular (EV, ivCD45.2^-^) and IV (ivCD45.2^+^) leukocytes (30). After a single Sp19F infection, lung EV B cells increased rapidly over 3 days before returning to baseline by day 10 (**Figure 1B**; gating strategy, **Supplementary Figure 1A**). In mice that received a second Sp19F infection on day 7 [to facilitate local antigen presentation in the lung (12, 31, 32)], we observed another, more substantial spike in B cell numbers after the second infection, peaking at day 10, and followed by contraction from day 14 to day 35. Thus, the second infection triggers robust B cell accumulation and is essential for B cell persistence in the lungs.

We leveraged machine learning to distinguish the types of B cells in the lungs after infection. Multidimensional data extracted via expert-driven gating of EV B cells at the day 10, day 14, and day 35 timepoints after 1 or 2 pneumococcal instillations were concatenated and clustered using unsupervised Louvain clustering via PhenoGraph algorithm (33). 21 unique subpopulations were resolved and overlaid onto the optimized parameter T-distributed stochastic neighborhood embedding (opt-SNE) projection of the multidimensional EV B cell cytometry data, revealing differences in Louvain cluster densities between timepoints (**Figures 1C, D**) (34). A heatmap of normalized marker expression for each subpopulation was generated for clustering and subset identification. Unsupervised hierarchical clustering dendrograms based on the relative mean fluorescence intensity of each marker enabled categorization of the 21 clusters into 6 major populations. The lowest numbered clusters (including 1 through 5), representing the most abundant cells, were IgD^+^ naïve B cells; a total of 10 clusters fell into this category (**Figure 1E**) (35, 36). 5 clusters were IgD^-^IgM^+^, two of which were further segregated by relative CD43 and B220 expression into subsets of IgM^+^ B2 and B1 cells (37). The remaining 5 clusters were IgD^-^IgM^-^ CSW B2 cells, which could be further segregated into non-GC B2 B cells and GC/pre-GC B2 cells based on their GL7 binding (28). Clusters expressing elevated GL7 were termed as GC (CD38^lo^) and pre-GC (CD38^hi^) B cells (38). Whether other notable B cell populations such as CD23^lo^CD21/35^+^ marginal zone (MZ) B cells and CD138^+^ TACI^+^ plasma cells were present were assessed in separate cytometry experiments (**Supplementary Figure 1B**). The lung was not observed to harbor MZ B cells, and plasma cells were present but likely underestimated due to the enzyme sensitivity of plasma cell defining markers and ongoing phenotyping challenges (39, 40). Specific markers for these populations were therefore excluded. These high dimensional clustering analyses guided the supervised gating strategies of various lung B cell subsets analyzed with flow cytometry throughout this study. Further, these data reveal previously underappreciated lung B cell population heterogeneity following respiratory exposures to pneumococcus.

**Figure 2:**
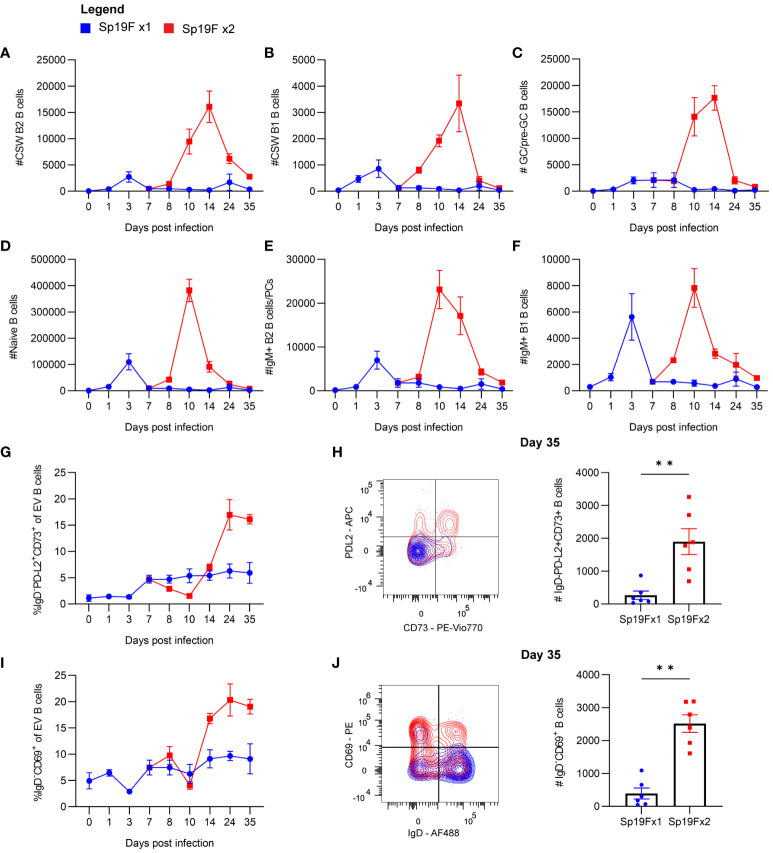
Kinetics of EV B cells after 1 or 2 self-limiting Sp infections. **(A)** Numbers of live EV CSW B2 (i.v.CD45.2^-^CD19^+^CD43^lo^B220^hi^IgD^-^IgM^-^), **(B)** CSW B1 (i.v.CD45.2^-^CD19^+^CD43^hi^B220^lo^IgD^-^IgM^-^), **(C)** GC/pre-GC (i.v.CD45.2^-^CD19^+^GL7^+^), **(D)** naïve (i.v.CD45.2^-^CD19^+^IgD^+^), **(E)** IgM^+^ B2 (i.v.CD45.2^-^CD19^+^CD43^lo^B220^hi^IgD^-^IgM^+^), **(F)** IgM^+^ B1 (i.v.CD45.2^-^CD19^+^CD43^hi^B220^lo^IgD^-^IgM^+^) B cells from lungs extracted at indicated timepoints following 1 (blue circles) or 2 infections (red squares) with Sp19F. **(G)** Proportion of live EV B cells that exhibit the memory B cell profile IgD^-^PD-L2^+^CD73^+^ at the indicated timepoints. **(H)** Representative flow plots of these populations from day 35 with overlays of Sp19F x1 (blue) and Sp19F x2 (red) groups, as well as numbers of these populations from each group. Mann Whitney test, **p<0.01. Analogous plots are depicted for EV B cells with the non-naive activation/residency markers IgD^-^CD69^+^ over the time course **(I)** and as flow plots with numbers at day 35 **(J)**. n=6 per group, 2 biological replicates.

To explore the population kinetics of different B cell subsets as they occupied the lung, we applied manual gating strategies (**Supplementary Figure 1A**) to lung EV B cells at all tested timepoints after 1 or 2 Sp19F infections from **Figure 1** and tracked quantities and frequencies of B cell subsets over time. We observed that CSW B2 B cells (**Figure 2A**), CSW B1 B cells (**Figure 2B**), and GC/pre-GC B cells (**Figure 2C**) numbers hardly deviated from baseline after only 1 infection, but all reached peak numbers at day 14 when a second infection had been administered. There were few to no CSW B1 and GC/pre-GC B cells found at later timepoints. Naïve B cells (**Figure 2D**), IgM^+^ B2 B cells (**Figure 2E**), and IgM^+^ B1 B cells (**Figure 2F**) acutely increased within the first 3 days after Sp19F re-exposure before numbers tapered. Other than innate-like IgM^+^ B1 B cells, each population experienced an exaggerated increase after repeat Sp19F instillation, whereas IgM^+^ B1 B cells populated the lung in nearly equal amounts 3 days after each exposure. In sum, we observe that the second infection leads to the accumulation of all EV B cell populations, with naïve and IgM^+^ B cells peaking earlier than GC/pre-GC and CSW B cells.

We previously used this model of anti-pneumococcal immunity to demonstrate that PD-L2^+^ MBCs are present in the lungs of experienced mice and confer anti-pneumococcal heterotypic immunity, likely via the local secretion of pneumococcus-specific antibodies (14). The presence of multiple MBC markers including PD-L2 and CD73 is associated with greater antibody affinity and likelihood to differentiate into antibody-secreting cells upon stimulation (41–43). To examine the kinetics of B_RM_ cell accumulation in the lung, we manually gated EV B cells bearing the memory phenotype IgD^-^PD-L2^+^CD73^+^ and determined their proportions among total EV B cells across the time course. After a single infection, the proportion of EV B cells that were non-naïve and expressing PD-L2 and CD73 remained between 0-5%, whereas numbers increased sharply 1 week after the second instillation and remained elevated (**Figure 2G**). EV B cells bearing memory markers were sparse in lungs that received only 1 exposure to Sp19F at the recovery time-point (**Figure 2H**). We similarly examined proportions of EV B cells bearing the non-naive activated/resident phenotype IgD^-^CD69^+^ after 1 and 2 pneumococcal infections. CD69 is a marker of resident memory in resting lymphocytes (9, 44), but is recognized as a marker of lymphocyte activation in non-resting states such as acutely after infection (45). Activated IgD^-^CD69^+^ B cells increased in proportion between days 10 and 14 and remained elevated thereafter (**Figure 2I**). As in the case of memory markers, EV B cells bearing the CD69 marker of residency in resting lungs were minimally present in the lungs that received only 1 Sp19F exposure by day 35 (**Figure 2J**). These data indicate that the second infection with Sp19F in this model system is essential for a transient burst of multiple B cell subsets in the lung immediately after the second infection, as well as for the establishment of lung B_RM_ cells in the recovered lung.

Among the Sp19F x2 groups, three timepoints stood out. Lungs at day 10 harbored the greatest overall number of B cells, lung CSW and pre-GC/GC B cells peaked at day 14, and lungs at day 35 contained EV B cells bearing markers of B cell memory and residency (12). We chose to next focus our examination of B cell populations to these three stages post-respiratory infection.

### B cell accumulation involves lymphocyte recirculation and local proliferation in the lungs

**Figure 3:**
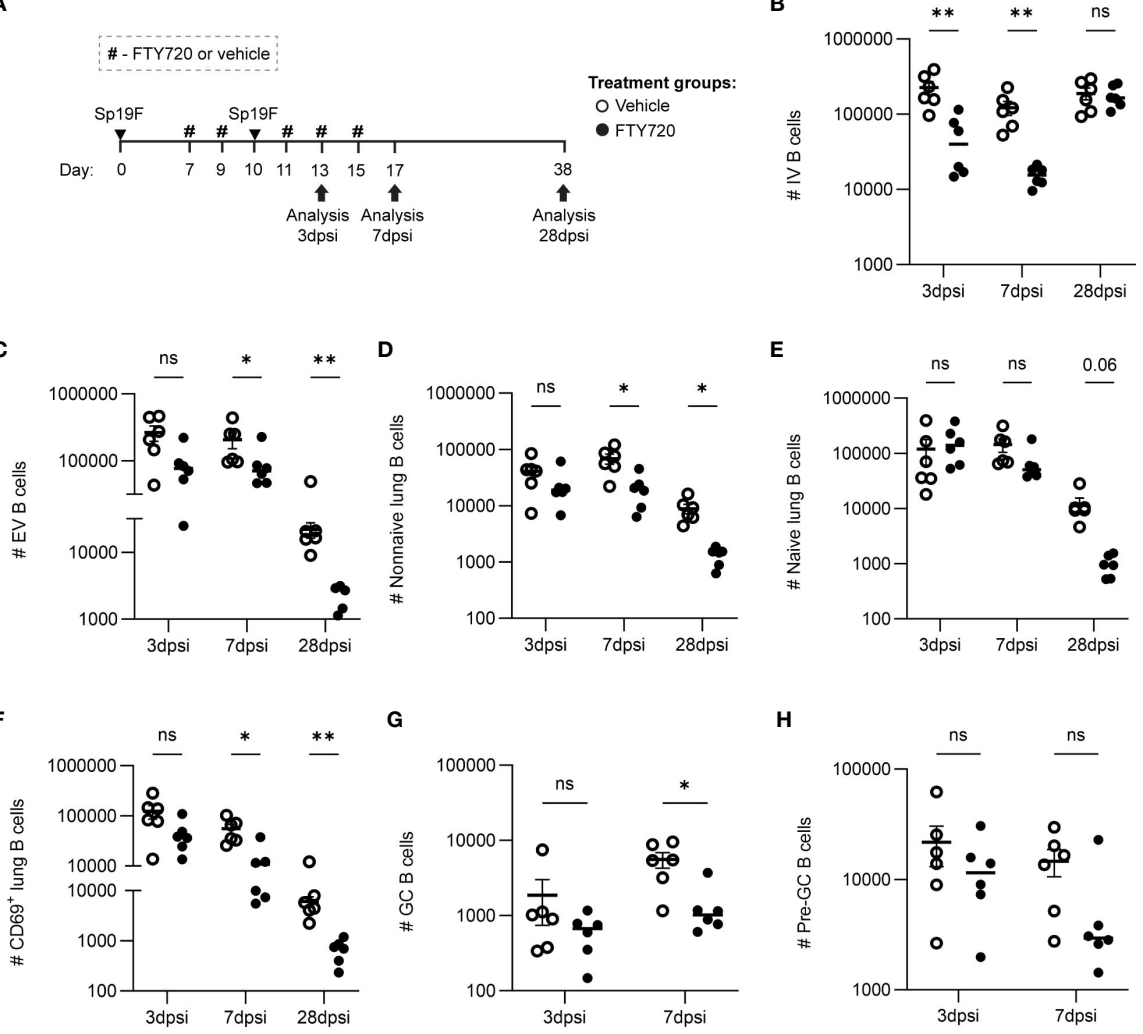
Lymphocyte recirculation during repeat Sp19F exposure is necessary for early antigen-activated B cell accumulation and lung EV B cell maintenance. **(A)** Before, during, and after a second Sp19F infection, mice were treated with i.p. FTY720 or saline vehicle at the timepoints indicated by the # symbol. Numbers of live **(B)** IV (i.v.CD45.2^+^CD19^+^), **(C)** EV (i.v.CD45.2^-^CD19^+^), **(D)** EV non-I (i.v.CD45.2^-^CD19^+^IgD^-^), **(E)** EV naïve (i.v.CD45.2^-^CD19^+^IgD^+^), and **(F)** EV CD69^+^ (i.v.CD45.2^-^CD19^+^CD69^+^) B cells were assessed at 2 dpsi, 7 dpsi, and 28 dpsi between vehicle (empty circles) and FTY720 (filled circles) treated groups. **(G)** GC B cells (i.v.CD45.2^-^CD19^+^GL7^+^CD38^lo^) and **(H)** pre-GC B cells (i.v.CD45.2^-^CD19^+^GL7^+^CD38^hi^) were enumerated at 2dpsi and 7dpsi only. Statistical significance was determined using multiple Mann-Whitney tests, *p<0.05, **p<0.01, ns, nonsignificant. n=5-6 per group, 2 biological replicates. y-axes are formatted in a log scale.

To determine if the recruitment of lymphocytes from draining lymph nodes was necessary for lung B cell accumulation after the second Sp19F infection, we administered the sphingosine-1-phosphate receptor 1 (S1P1R) inhibitor FTY720 or vehicle treatment intraperitoneally (i.p.) before, during, and after the second infection (**Figure 3A**) and used flow cytometric analysis to quantify B cell populations at 3 days post second infection (dpsi; corresponding to day 10 in **Figures 1**, **2**), 7dpsi (corresponding to day 14), and 28dpsi (corresponding to day 35). This treatment schedule allowed effectors to be established normally in the lung and peripheral secondary lymphoid organs after the first infection but prevented lymphocyte egress from draining lymph nodes upon the second Sp19F exposure in FTY720-treated mice (46). A less comprehensive surface marker panel was used to analyze B cell populations in this experiment as we were predominantly interested in determining whether increases in lung EV non-naive B cells would still occur in the presence of a lymphocyte egress inhibitor, whether local GC reactions would take place (indicated by the presence of GC and pre-GC B cells), and whether antigen-activated B cells accumulate in the lung (indicated by CD69 at acute timepoints). Suppression of lymphocyte egress expectedly reduced circulating B cell numbers during the FTY720 treatment (**Figure 3B**). This treatment significantly reduced the total numbers of lung B cells by 28dpsi, but differences were more subtle at earlier timepoints (**Figure 3C**). Lung non-naive (IgD^-^) B cells were significantly reduced from 7dpsi onwards in the FTY720-treated groups (**Figure 3D**), whereas lung naïve (IgD^+^) B cells trended towards decrease but were not statistically different at any timepoint tested (**Figure 3E**). CD69^+^ B cells were significantly reduced at 7dpsi and 28dpsi in the FTY720-treated groups, but also exhibited a trending (p=0.06) decrease acutely at 3dpsi relative to vehicle-treated mice (**Figure 3F**). Similarly, GC B cells (GL7^+^CD38^lo^) were found to decrease significantly at 7dpsi with FTY720 treatment (**Figure 3G**), while pre-GC B cells (GL7^+^CD38^hi^) did not (**Figure 3H**), suggesting that the initiation of local GC reactions also requires intact lymphocyte circulation during the second exposure to pneumococcus. GC and pre-GC B cell populations are scarce at 28dpsi in the Sp-experienced lung and were thus excluded from data collection at the lattermost timepoint. These data suggest that the immune cells established initially in the lung following the first Sp19F infection were insufficient in driving the phenotypic changes in the lung B cell populations associated with B_RM_ cell development upon subsequent pneumococcal exposure, and that continuous recruitment from the periphery during antigen exposure is necessary for lung EV B cell maintenance.

Local secretion of the follicular chemokine C-X-C motif chemokine ligand 13 (CXCL13) is necessary to recruit naïve CXCR5^+^ B cells from the periphery to form lung ectopic GCs, iBALT, and lung MBCs after IAV infection (22). On the other hand, *Pseudomonas aeruginosa* infection induces iBALT with CXCR4-expressing B cells attracted to CXCL12 rather than CXCL13 (21). The secretion of these chemokines is triggered by IL-17 and serves to attract B cells into follicles and stabilize contact with T follicular helper (T_FH_) cells (47). Outside of homing to follicles, CXCL13 also has a role in B1 B cell compartmentalization in the pleura and peritoneum (48). Although pneumococcal infections do not support persisting iBALT, we wanted to test whether a conserved mechanism for B cell recruitment existed in this Th17 cell-driven model of anti-bacterial immunity (12). CXCL13 and CXCL12 protein levels in whole lung homogenates were measured using ELISAs at various time points after Sp19F infections (**Supplementary Figure 2A**). From days 8-10, CXCL13 protein increased significantly after 2 Sp19F infections and correlated with the accelerated increase in EV B cell numbers observed in **Figures 1**, **2**. In contrast, CXCL12 levels did not change. Although this implicated CXCL13 as a potential driver of B cell recruitment and follicular B cell proliferation in the lung, administration of an anti-CXCL13 blocking antibody did not impact B_RM_ cell quantities or proportions when administered i.p. and intranasally (i.n.) at the same timepoints defined in **Figure 3A** (**Supplementary Figures 2B–D**) (49). Redundant mechanisms besides CXCL13 likely serve to recruit B cells to the lung, and this chemokine may be less important in models that do not support lasting mature iBALT. Of parallel note, the CXCL13/CXCR5 axis has been implicated in B cell homing to the central nervous system in several neuroinflammatory diseases, but it is dispensable for B cell recruitment, antiviral antibody responses, and overall disease course in alphavirus encephalitis, despite strong induction of CXCL13 (50). These studies highlight pathogen-dependent variations in B cell responses that can occur across tissues.

**Figure 4:**
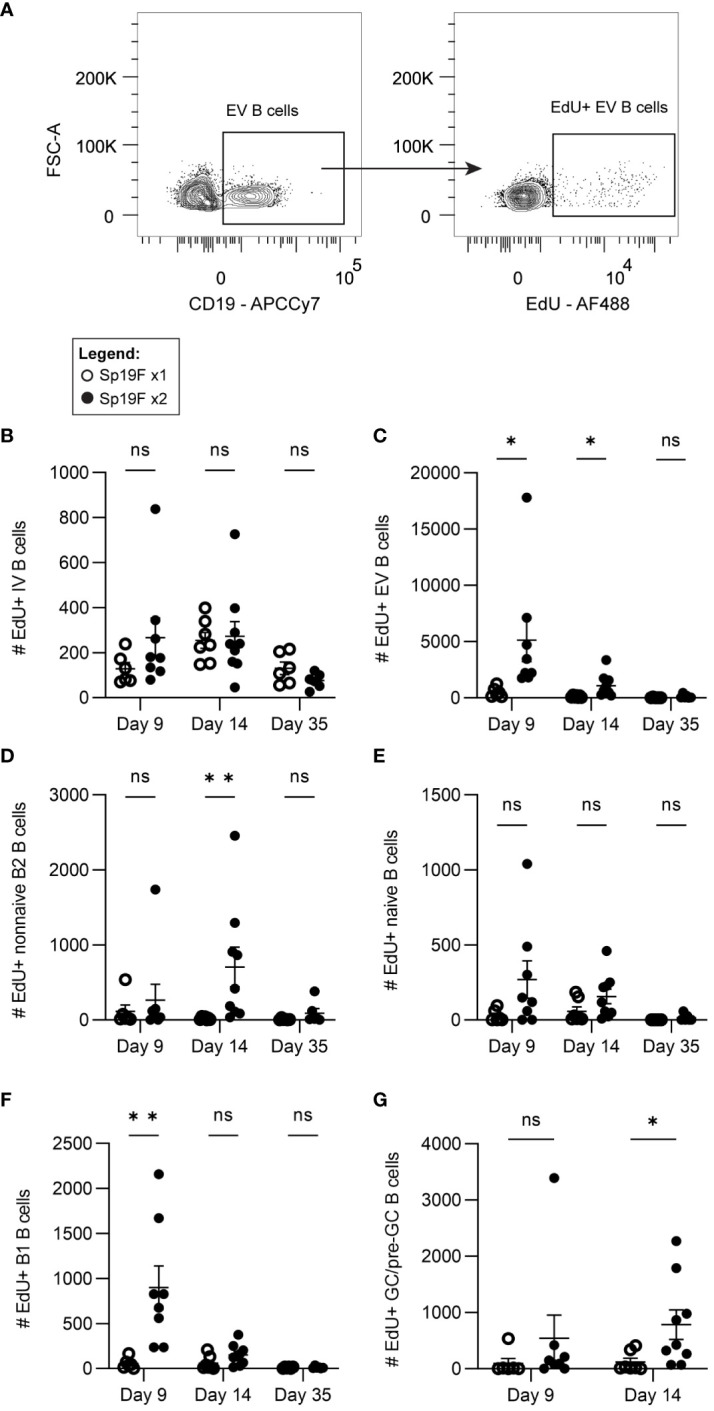
Lung EV B cells exposed to 2 infections undergo self-renewal. **(A)** Representative flow plots demonstrating manual gating of EdU^+^ EV B cells, gated based on fluorescence minus one controls. Numbers of EdU^+^ **(B)** IV (i.v.CD45.2^+^CD19^+^), **(C)** EV (i.v.CD45.2^-^CD19^+^), **(D)** non-naive B2 (i.v.CD45.2^-^CD19^+^IgD^-^CD43^lo^B220^hi^), **(E)** naïve (i.v.CD45.2^-^CD19^+^IgD^+^), **(F)** B1 (i.v.CD45.2^-^CD19^+^CD43^hi^B220^lo^), **(G)** and GC/pre-GC (i.v.CD45.2^-^CD19^+^GL7^+^) B cells at day 9, day 14, and day 35 after 1 (empty circles) or 2 (filled circles) Sp19F infections. n=6-9 per group, 3 biological replicates. Analyzed with multiple Mann-Whitney tests, *p<0.05, **p<0.01, ***p<0.001. ns, not significant.

While the FTY720 data suggest that B cell recirculation is required for the maximal accumulation of B cells observed after 2 doses of Sp19, they do not preclude an additional contributory role for B cell proliferation in the lungs. Given the findings in **Figure 3**, it is possible that S1P1R inhibition prevented other lymphocytes from entering the lung, such as CD4^+^ T cells, which could facilitate B cell proliferation *in situ* despite a lack of mature GC structures or known T_FH_ cells. This would be in line with recent findings using an IAV vaccination model and adoptive transfer strategy to determine that the formation of certain CD4^+^ T cell effectors and memory populations require cognate antigen/antigen-presenting cell exposure 6-8 days after initial antigen exposure (51). This included lung T_FH_ cells, which have been demonstrated in mature iBALT-inducing models, but thus far remain elusive in Sp infection models (32, 52–54). To capture only the cells in the lung that are dividing over the short period after infection, rather than those that had proliferated earlier in lymph nodes, we administered 5-ethynyl-2′-deoxyuridine (EdU) i.p. to mice 4 hours prior to euthanasia on day 9, day 14, or day 35 and used flow cytometric analysis to enumerate actively proliferating B cells. This was accomplished by fluorescently tagging EdU^+^ cells via copper-catalyzed click reactions (**Figure 4A**; gating strategy **Supplementary Figure 3A**). Panel breadth was limited due to the interference of copper with certain fluorophores. Quantities of EdU^+^ IV B cells were relatively low and unchanged between Sp19F x1 and Sp19F x2 groups (**Figure 4B**). Among EV B cells, EdU was detected in groups that received 2 infections at each tested timepoint, but not in those that received only 1 exposure to Sp19F (**Figure 4C**). By day 35, the Sp-experienced lung is at a resting immune state and B cell proliferation is expectedly minimal. After two Sp19F infections, EdU is incorporated into proliferating non-naive B2 B cells predominantly at day 14 (**Figure 4D**). EV naive B cells did not exhibit significant increases in proliferation at any timepoint (**Figure 4E**). EdU^+^ B1 B cells were only detected at day 9, reflecting their properties as proliferating acute responders (**Figure 4F**). Finally, as with non-naive B2 B cells, B cell proliferation was significantly increased among lung pre-GC/GC B cells (**Figure 4G**). These findings demonstrate that the pneumococcus-experienced lung undergoes changes that can support non-classical GC driven proliferation of EV B cells after repeat exposure to T-independent (TI) and T-dependent (TD) antigens.

### The early accumulation and activation of B cells in infected lungs relies on CD4^+^ cells

**Figure 5:**
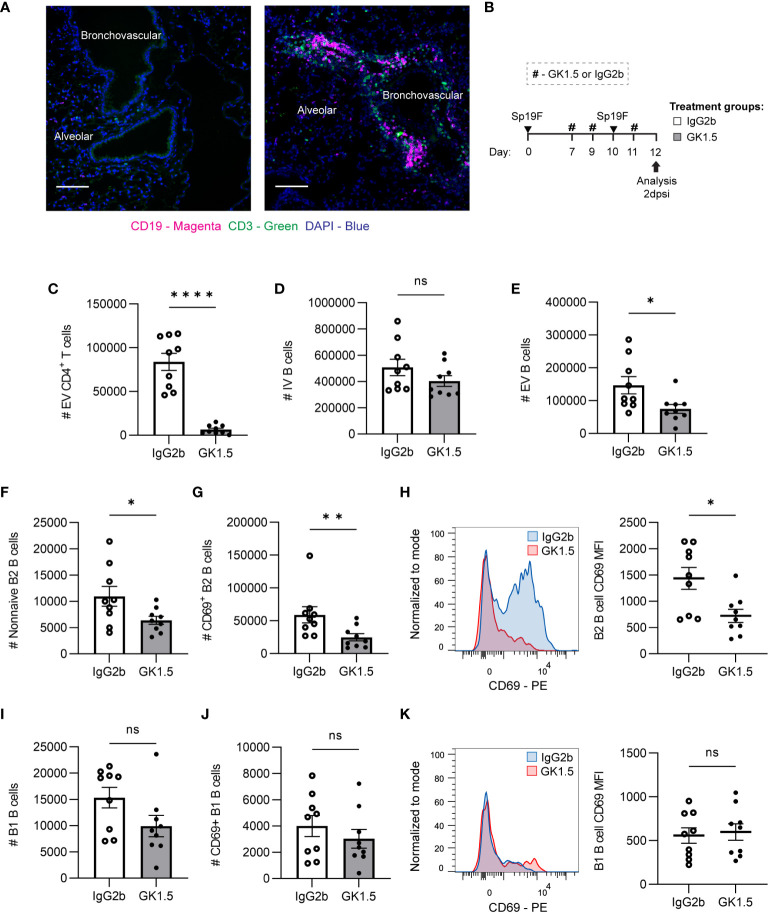
CD4^+^ cell depletion during antigen presentation in the lung affects early EV B cell accumulation. **(A)** Representative IF image of naïve mouse and Sp-experienced mouse lung BV bundles and alveolar regions stained for CD19 (magenta), CD3 (green), and DAPI (blue) at day 0 and at day 9 (2 dpsi). Scale bars signify 100 μm. **(B)** GK1.5 vs. IgG2b isotype control was administered i.p. and i.n. to B6 mice before, during, and after the second infection with Sp19F at the timepoints indicated by the # symbol. Mice were euthanized 2 dpsi for flow cytometric analysis. **(C)** Enumeration of live EV CD4+ T (i.v.CD45.2^-^CD4^+^CD3^+^), **(D)** IV B (i.v.CD45.2^+^CD19^+^), **(E)** EV B (i.v.CD45.2^-^CD19^+^), **(F)** non-naive B2 B (i.v.CD45.2^-^CD19^+^IgD^-^CD43^lo^B220^hi^), and **(G)** CD69^+^ B2 B (i.v.CD45.2^-^CD19^+^IgD^-^CD43^lo^B220^hi^CD69^+^) cells after treatment with GK1.5 (white bars) or IgG2b (grey bars). **(H)** Representative overlaid EV B2 B cell CD69 MFI plots normalized to mode from mice treated with GK1.5 (red) vs. IgG2b (blue), with accompanying individual sample MFIs. **(I)** Enumeration of EV B1 B (i.v.CD45.2^-^CD19^+^CD43^hi^B220^lo^) and **(J)** EV CD69^+^ B1 B (i.v.CD45.2^-^CD19^+^CD43^hi^B220^lo^CD69^+^) cells. **(K)** Representative overlaid EV B1 B cell CD69 MFI plots normalized to mode from mice treated with GK1.5 (red) vs. IgG2b (blue), with accompanying individual sample MFIs. n=9 per group, 3 biological replicates. Mann-Whitney tests; *p<0.05, **p<0.01, ****p<0.0001; ns, not significant.

Although lacking mature tertiary lymphoid structures, known lung T_FH_ cells, or dependence on the follicular chemokine CXCL13, we observed proliferative B2 and GC B cells *in situ* in the lung after two Sp19F infections, suggesting that follicular signaling such as CD40-CD40L interactions between T and B cells may take place locally after the second exposure to Sp19F (38, 55). This led us to question the extent to which B cells in this model may depend on CD4^+^ T cell interactions for establishment in the lung. At 2dpsi, B cells marked by CD19 and T cells marked by CD3 were observed both as aggregates around bronchovascular areas as well as across alveoli (**Figure 5A**). Although found to be colocalized around bronchovascular bundles, the patterns of B and T cells observed at this timepoint were not consistent with those of organized follicular structures and were likely instead reflective of acute lymphocyte infiltration.

Since we observed B and T cells together in the lung at 2 dpsi, we tested whether CD4^+^ cells impacted initial B cell accumulation after the second Sp exposure. To do so, we administered the anti-CD4 antibody clone GK1.5 or IgG2b as an isotype control to C57BL/6J (B6) mice as previously described (12), using the treatment timepoints depicted in **Figure 5B**. This allowed the usual establishment of lymphocytes in the lung and periphery after the initial infection, but the second exposure to antigen occurred after CD4^+^ cell depletion. Lungs were extracted at 2 dpsi for flow cytometric analysis of early lung B cell populations. Depletion of CD4^+^ cells (**Figure 5C**) just before the second Sp19F infection did not impact IV blood B cells but diminished EV lung B cells (**Figures 5D, E**). Non-naive B2 B cells and especially CD69^+^ acutely activated B2 B cells were significantly reduced among the GK1.5-treated mice, relative to isotype control (**Figures 5F, G**). Not only were there fewer CD69^+^ B cells, but the CD69 median fluorescence intensity (MFI) levels were also lower on those expressing this activation marker in the GK1.5-treated group, serving as evidence for decreased activation during acute infection (**Figure 5H**). On the other hand, CD4^+^ cell depletion did not significantly affect B1 B cells, CD69^+^ B1 B cells, or expression of CD69 on B1 B cells 2 days post-infection (**Figures 5I–K**). This may be because B2 B cells are equipped with adaptive B cell receptors (BCRs) that respond to TD protein antigens and have the capacity to undergo follicular reactions, whereas B1 BCRs are germline-encoded with reactivity to TI antigens including pneumococcal capsular polysaccharide and cell wall phosphorylcholine (29, 56). These findings demonstrate that the initial accumulation of antigen-activated B2 B cells (whether via peripheral recruitment and/or *in situ* proliferation) following repeat antigen exposure exhibits some reliance on CD4^+^ cells, while B1 B cell accumulation and activation do not.

### The establishment of lung B_RM_ cells requires CD4^+^ cells during infection

**Figure 6:**
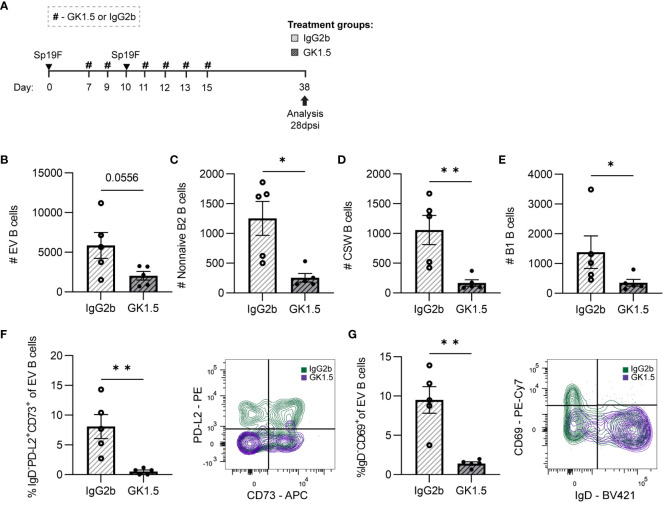
CD4^+^ cell depletion during second Sp19F infection abrogates lung B_RM_ cells. **(A)** GK1.5 vs. IgG2b isotype control was administered i.p. and i.n. to B6 mice before, during, and after the second infection with Sp19F at the timepoints indicated by the # symbol. Mice were euthanized at 28 dpsi for flow cytometric analysis. **(B)** Enumeration of live EV B (i.v.CD45.2^-^CD19^+^), **(C)** non-naive B2 B (i.v.CD45.2^-^CD19^+^IgD^-^CD43^lo^B220^hi^), **(D)** CSW B (i.v.CD45.2^-^CD19^+^IgD^-^IgM^-^), and **(E)** B1 B (i.v.CD45.2^-^CD19^+^CD43^hi^B220^lo^) cells after treatment with GK1.5 (white bars with grey stripes) or IgG2b (grey bars with black stripes). Representative flow plots depicting memory (IgD^-^PD-L2^+^CD73^+^), **(F)** and resident (IgD^-^CD69^+^), **(G)** EV B cell populations with overlaid IgG2b (green) and GK1.5-treatment (purple) conditions. Associated graphs depicting each population as a percentage of EV B cells for individual samples are provided. n=5-6 per group, 2 biological replicates. Mann-Whitney tests; *p<0.05, **p<0.01; ns, not significant.

Since we observed differences in lung B cell subsets 2dpsi following CD4^+^ cell depletion but did not see complete erasure of initially formed lung B cell subsets 2 dpsi, we wanted to examine how the removal of CD4^+^ cell interactions during the second exposure to Sp19F impacted the maintenance of different lung B cell subsets and the establishment of B_RM_ cells in the Sp-experienced lung. We have previously observed loose aggregates of B and T cells at this timepoint after Sp19F infections and determined that these were not mature follicular structures based on absent peripheral node addressin staining (14). The presence of B_RM_ cells in a model lacking mature iBALT structures and known T_FH_ cells raised the question of whether B_RM_ cell formation necessitated interactions with CD4^+^ cells during repeat antigen exposure. To test this, we administered GK1.5 or isotype control throughout the second exposure to Sp19F as in **Figure 5**, extending the timeline to 28 dpsi (**Figure 6A**).

While circulating IV B cells were again indifferent to CD4^+^ cell depletion (**Supplementary Figures 4A, B**), we observed a trending decrease in total EV B cells (**Figure 6B**). Both non-naive B2 B cells and CSW B cells were decreased in number (**Figures 6C, D**), demonstrating that CD4^+^ cells are important for the establishment of these cells in the recovered lung. In addition, we were surprised that B1 B cell quantities diminished among the GK1.5-treated mice as well (**Figure 6E**). The significant decrease in lung B1 B cells suggests that they may directly or indirectly rely on CD4^+^ cells or other B2 B cells for maintenance in the lung.

B_RM_ cells with phenotypes associated with memory (IgD^-^PD-L2^+^CD73^+^; **Figure 6F**) and residency (IgD^-^CD69^+^; **Figure 6G**) were completely abolished in the GK1.5-treated group. The frequencies of naïve and non-resident EV B cells were unchanged between treatment groups (**Supplementary Figure 4B**), indicative of a selective reliance on CD4^+^ cells for the formation and/or maintenance of B_RM_ cell populations in the lung. At resting timepoints, CD69 serves as a marker of lymphocyte residency rather than activation, therefore expression measured via MFI was not assessed as in the acute timepoints (45, 57). Taken together, these results demonstrate that lung BRM cells require CD4^+^ cells after recovery from pneumococcal infections.

The shift from IgM to other BCR isotypes resulting from class switch recombination is predominantly facilitated by specific cytokine and CD40 signals received by T helper cells, but can also occur without T cell help (58, 59). To assess the function of IgM^+^ and CSW antibody-secreting cells in CD4^+^-depleted experienced mice, we used bacterial whole-cell ELISAs to measure relative levels of Sp-reactive airspace antibodies in the bronchoalveolar lavage fluid (BALF) of the mice treated with GK1.5 vs. IgG2b. As these mice were experienced but in a resting state, we interpreted antibodies measured in the BALF as being sourced from baseline antibody-secreting cell activity in the lung, rather than from acutely formed antibody-secreting cells responding to antigen re-exposure. Therefore, we could use this as a proxy to determine whether these interventions during the second exposure to Sp19F impacted the formation or retention of lung plasma cells with serotype-independent reactivity against pneumococcal proteins, which we have previously demonstrated as a likely protective mechanism conferred by lung B_RM_ cells in this model (14). In agreement with the apparent dependence on CD4^+^ cells for the formation or maintenance of CSW B cells, we found that CD4^+^ cell depletion with GK1.5 during the second infection resulted in significantly decreased levels of airspace IgG and IgA reactive to acapsular Sp3 and a trending decrease in IgM (**Supplementary Figure 4C**). These data suggest that the formation or maintenance of lung humoral immunity depends on the presence of CD4^+^ cells while antigen is being presented locally in the lung.

### CD40L-CD40 interactions during infection are required for lung B_RM_ cells

**Figure 7:**
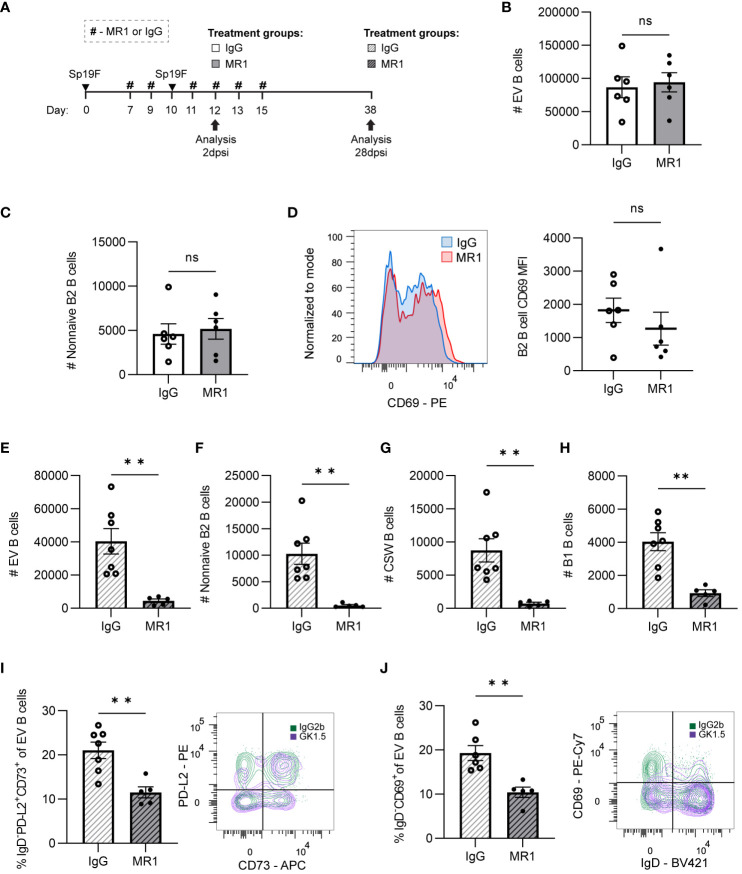
CD40L is dispensable for initial lung B cell accumulation but is required for lung B cell maintenance and the establishment of B_RM_ cells. **(A)** MR1 vs. IgG isotype control was administered i.p. and i.n. to B6 mice before, during, and after the second infection with Sp19F at the timepoints indicated by the # symbol. Mice were euthanized at 2 dpsi and 28 dpsi for flow cytometric analysis. Enumeration of live **(B)** EV B and **(C)** non-naive B2 B cells (i.v.CD45.2^-^CD19^+^IgD^-^CD43^lo^B220^hi^) at 2 dpsi after treatment with MR1 (white bars) or IgG (grey bars). **(D)** Representative overlaid EV B2 B cell CD69 MFI plots normalized to mode from mice treated with MR1 (red) vs. IgG (blue), with accompanying individual sample MFIs. **(E)** Enumeration of live EV B (i.v.CD45.2^-^CD19^+^), **(F)** non-naive B2 B (i.v.CD45.2^-^CD19^+^IgD^-^CD43^lo^B220^hi^), **(G)** CSW B (i.v.CD45.2^-^CD19^+^IgD^-^IgM^-^), and **(H)** B1 B (i.v.CD45.2^-^CD19^+^CD43^hi^B220^lo^) cells at 28 dpsi after treatment with MR1 (white bars with grey stripes) or IgG (grey bars with black stripes). Representative flow plots depicting memory (IgD^-^PD-L2^+^CD73^+^), **(I)** and resident (IgD^-^CD69^+^), **(J)** EV B cell populations with overlaid IgG (green) and MR1-treatment (purple) conditions. Associated graphs depicting each population as a percentage of EV B cells for individual samples are provided. n=5-6 per group, 2 biological replicates. Mann-Whitney tests; **p<0.01; ns, not significant.

As CD40L blockade has commonly been used to demonstrate T-dependent processes in B cells (60), and because we observed decreased B2 B cell and CSW B cells in CD4^+^ cell-deplete mice, we used the anti-CD40L blocking antibody (MR1) or isotype control (IgG) to test if the T-dependent contributions to B cell population establishment could be associated with CD40 signaling. As in the CD4^+^ cell depletion experiments, we tested whether MR1 administered through the second infection period impacted the initial and/or established lung B cell populations (**Figure 7A**). Interestingly, CD40L blockade did not have any impact on cell numbers (**Figures 7B, C**; **Supplementary Figure 5A**) or activation state (**Figure 7D**) of early B cell subsets found at 2 dpsi. Synthesis of findings depicted here with those of **Figure 5** suggests that CD4^+^ cells mediate acute B cell accumulation and activation in the lung independently of CD40-CD40L interactions.

At 28 dpsi, consequences of administering CD40L blockade during local antigen presentation were evident. Although IV circulating B cells were intact, EV lung B cell numbers were diminished when CD40L had been blocked during infection (**Figure 7E**; **Supplementary Figure 5B**). Non-naive B2 B cells (**Figure 7F**) and CSW B cells (**Figure 7G**) were virtually eliminated due to the prior MR1 treatment. We again observed significant decreases in B1 B cells (**Figure 7H**), which do not typically require CD40-CD40L interactions (61). As in GK1.5-treated mice, the proportion of B_RM_ cells with phenotypes associated with memory (IgD^-^PD-L2^+^CD73^+^; **Figure 7I**) and residency (IgD^-^CD69^+^; **Figure 7J**) were significantly lower in the MR1-treated group than in the IgG-group. Thus, while CD40L-CD40 is not essential for the early CD4^+^ cell-dependent accumulation and activation of B cells during infection, these data implicate CD40-CD40L interactions as the mechanism by which CD4^+^ cells contribute to pan-B cell maintenance and B_RM_ generation in the lung.

Class switch reactions can occur via T helper and CD40L-dependent or independent mechanisms, with the latter providing low-affinity antibodies with specialized effector functions early in the immune response. IgA-secreting plasma cells and T-independent class switch reactions from IgM to IgA among intestinal B cells, but not colonic B cells, were previously found to be intact in CD40 knockout mice that are unable to form GCs (59). To assess the potential effect of CD40L-blockade on the formation and/or maintenance of lung antibody-secreting cells, BALF was collected at 28 dpsi for analysis of Sp-reactive airspace antibodies in the MR1-treated vs. IgG-treated mice (**Figure 7A**). MR1-mediated blockade of CD40L resulted in decreased Sp-reactive IgG, but IgM and IgA were left intact (**Supplementary Figure 5C**). Given that B cells were also globally affected by the loss of CD4^+^ cells and CD40L blockade, it is likely that the reduced supply of CSW B cells to become IgG and IgA-secreting antibody-secreting cells played a role in the observed differences in antibody-secreting cell activity, but it is also possible that CD4^+^ cells could contribute to a milieu that can support antibody-secreting cells.

### Sp-experienced B_RM_ cells do not require CD4^+^ T cell help for memory recall

We next questioned whether CD4^+^ T cells were necessary for optimal B_RM_ cell recall function. Reactivation of MBCs during secondary exposure to antigen was thought to require primed T cell help (24). Recent studies utilizing respiratory IAV infection described the induction of T_FH_-like CD4^+^ T cells expressing BCL6 and/or FR4 in the lung, and showed that deletion of *Bcl6* at later resting timepoints, impaired local antibody production upon rechallenge (53, 54).

**Figure 8:**
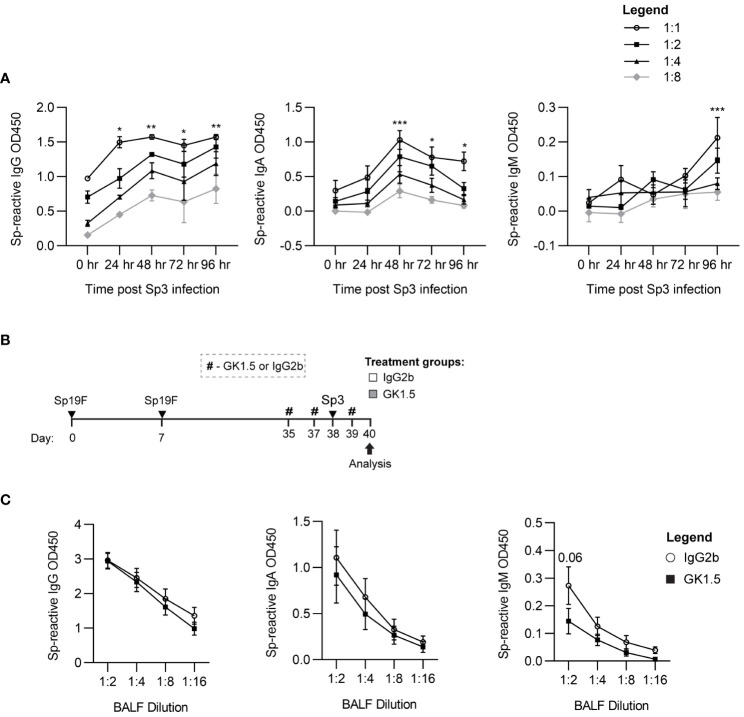
CD4^+^ cells are not required for the recall function of Sp-reactive BRM cells. **(A)** Acapsular Sp3-reactive IgG, IgA, and IgM titers in BALF from Sp19F-experienced mice in 24 hour increments after respiratory challenge infection with Sp3. n = 3-6 per timepoint across 2 independent experiments. 2way ANOVA; *p<0.05; **p<0.01; ***p<0.001. **(B)** Experimental timeline to generate anti-pneumococcal immunity in B6 mice followed by treatment with CD4 cell-depleting GK1.5 or IgG2b isotype control antibody 72 and 24 hours before and 24 hours after Sp3 challenge as indicated by the # symbol. **(C)** Acapsular Sp3-reactive IgG, IgA, and IgM titers in BALF from Sp19F-experienced mice treated with GK1.5 vs. IgG2b during Sp3 challenge were measured via whole-cell pneumococcal ELISAs 48 hpi. n = 5-6 per group across 2 independent experiments. 2way ANOVA.

To test whether CD4^+^ T cell help was required for lung MBC heterotypic recall responses to Sp3 challenge, we treated Sp-experienced B6 mice with the CD4^+^-cell depleting antibody GK1.5 or IgG2b isotype control before and during respiratory Sp3 infection to deplete circulating and parenchymal CD4^+^ cells (**Figure 8A**) (12). BALF was collected 48 hpi for ELISA quantitation of secreted crossreactive antibodies recognizing acapsular Sp as we have previously observed differences from baseline at this timepoint in Sp3-challenged experienced mice for Sp-reactive IgG and IgA (**Figure 8B**). We did not observe differences in airspace Sp-reactive IgG and IgA titers between the GK1.5- and isotype-treated groups at 48 hpi. Sp-reactive IgM was found to have a trending decrease in CD4^+^ T cell-depleted mice compared to isotype control-treated mice (**Figure 8C**), although airspace Sp-reactive IgM levels are not significantly elevated above baseline at this timepoint. As IgM secretion is associated with acute B cell activation, the trending differences seen in airway Sp-reactive IgM may reflect indirect changes in the inflammatory milieu secondary to CD4^+^ cell absence that prevented the recruitment or activation of acutely responding B cells (56, 62).These findings suggest that, like systemic MBCs, T cell dependence for lung B_RM_ cell reactivation is likely context-dependent. Whether the lack of access to follicular support is a factor in T-independent lung B memory recall will require more studies of B_RM_ cells in different infectious and autoimmune contexts.

### Lung B cells occur in bronchovascular bundles and across alveolar septae

**Figure 9:**
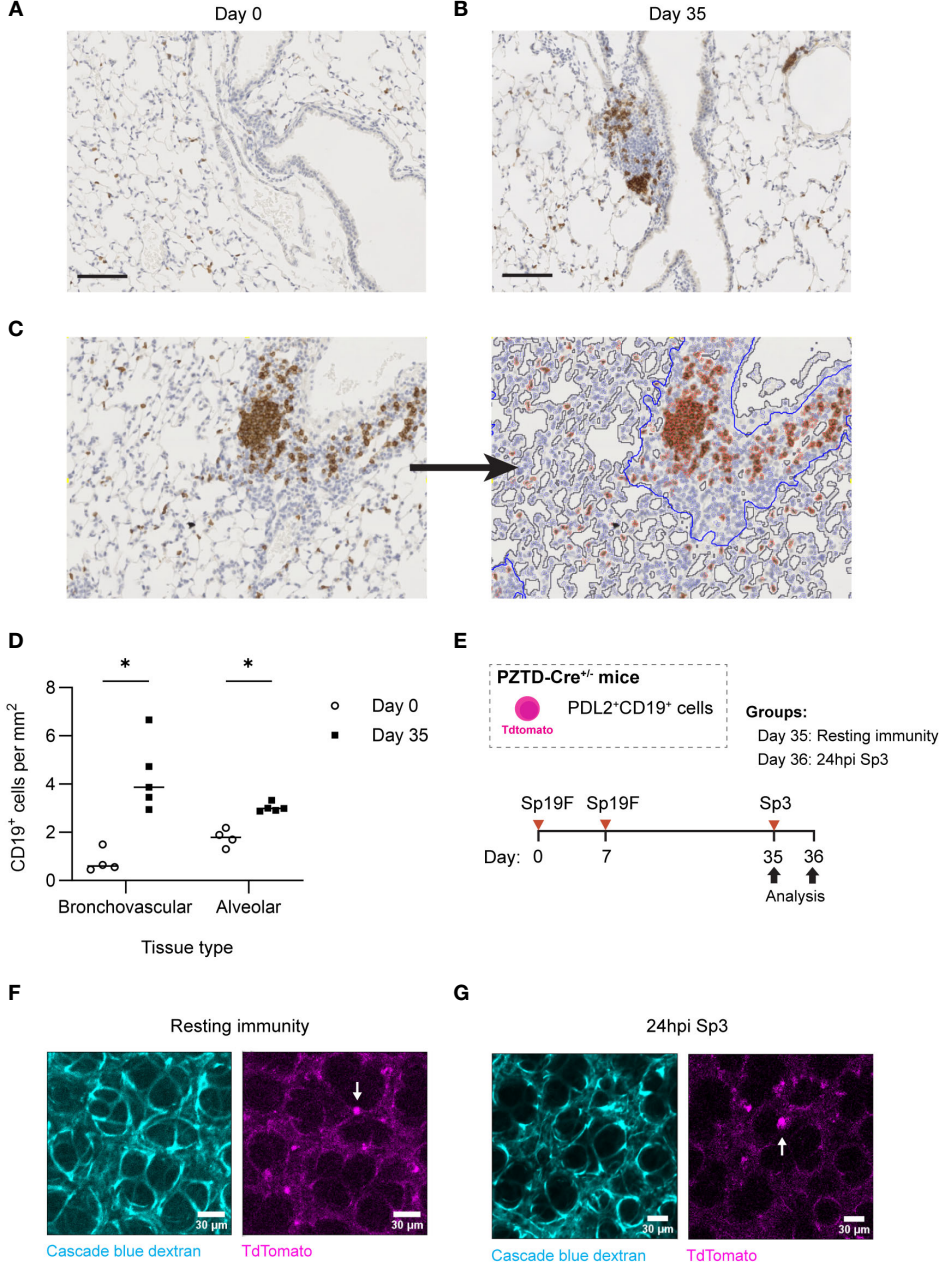
Lung B and B_RM_ cells localize to BV bundles and across alveoli in the Sp-experienced lung. **(A)** Representative immunohistochemical staining of CD19^+^ cells extracted from B6 mouse lungs infected twice with Sp19F i.t. at day 0 and **(B)** day 35. Scale bars represent 50 μm. **(C)** Example image tile before and after markup applied in QuPath. BV bundles and associated infiltrates are outlined in blue. Airway and vessel lumens are outlined in grey for exclusion. Positively and negatively stained cells are traced in red and blue, respectively. **(D)** Quantification of the numbers of positively stained CD19^+^ cells per area (mm^2^) of alveolar or bronchovascular tissue at each timepoint (day 0 - open circles, day 35 - filled circles) after infection. Slides for blinded analysis were selected at random from 3 independent experiments, totaling 4-5 mice per timepoint across replicates. Multiple Mann-Whitney tests, *p<0.05. **(E)** Experimental timeline to generate Sp-experience in CD19-Cre PZTD mice followed by challenge with Sp3 in recovered mice. **(F)** Lung and heart blocks were extracted from Sp-experienced CD19-Cre PZTD mice for live *ex vivo* imaging; screen captures before and **(G)** after 24 hpi with Sp3 are presented here. Pulmonary vasculature is marked with fluorescent Cascade blue dextran (blue). The TdTomato endogenous reporter marks CD19^+^PD-L2^+^ cells (magenta), examples denoted by white arrows.

In lungs recovered from pneumococcus, B cells are most prominent in loose leukocyte aggregates within the bronchovascular bundles (14). However, after IAV infection, lung B_RM_ cells are also numerous and active throughout the alveolar septae (16). Given that CD4^+^ cells were not required for memory recall, we hypothesized that B_RM_ cells might also be found outside of lymphocyte aggregates in lungs recovered from pneumococcus. We first sought to quantify B cells in these different anatomic compartments of lungs recovered from pneumococcal infections. At day 0, alveoli were patent, and the lung was without infiltrates or obvious immune cell aggregates. B cells were diffusely spread throughout the alveolar septae, but we were unable to determine whether they were intravascular or extravascular (**Figure 9A**). At day 35, we observed clusters of CD19^+^ cells within loose lymphoid aggregates in the bronchovascular compartment, with preserved alveolar architecture akin to that observed in uninfected lungs (**Figure 9B**).

CD19^+^ cells were quantified in peri-bronchovascular and alveolar compartments in a semi-automated manner using the imaging analysis platform QuPath (63) (**Figure 9C**). This allowed us to normalize differences in anatomic phenomena and exclude luminal airspaces across sections from different mice that may otherwise bias B cell quantification. Across lung sections, a mean of 77% of tissue was classified as alveolar while 33% was bronchovascular. Our analysis revealed that the number of CD19^+^ cells per square millimeter (mm^2^) of alveolar tissue in the experienced mice increased significantly compared to numbers in naïve uninfected lungs at day 0 (**Figure 9D**). This may represent increases in EV B cells that populate alveolar areas after Sp experience in addition to a consistent number of IV B cells circulating through the lung. Changes in B cell density were more pronounced between naïve and experienced mice in the bronchovascular compartment, which saw a >4-fold increase in CD19^+^ cells per mm^2^ between naive and experienced lungs. Combining the relative densities of B cells in each tissue-type with the fractions of lung comprised of each tissue type, we approximate that the increased B cells in the recovered lung are similarly numerous across the tissue types (distributed as 55% in the alveolar septae and 45% in bronchovascular regions).

We previously used the transgenic CD19-Cre PD-L2-ZsGreen-TdTomato-diphtheria toxin receptor (PZTD) mouse line, which operates as both an inducible-depletion system and as a fluorescent reporter to show that 28 days after 2 pneumococcal infections, PD-L2^+^ B cells are only present in the EV spaces of the lung, where they function as protective B_RM_ cells (14, 64). This system therefore allows us to differentiate the anatomic location of EV B cells in the lungs, if we localize these PD-L2^+^ B_RM_ cells in the Sp-experienced lung to distinct anatomic sites. After IAV infection, the B cells in the alveolar region are motile when examined using precision cut lung slices (16). Therefore, in the present study, we used *ex vivo* confocal microscopy in the crystal ribcage (65) to visualize TdTomato^+^ cells in the peripheral parenchyma as PD-L2^+^ B_RM_ cells. We generated heterotypic immunity in these mice to allow for the tracking of PD-L2^+^CD19^+^ cells with TdTomato (**Figure 9E**). Live imaging of CD19-Cre PZTD lungs with Sp-experience revealed that the PD-L2^+^ memory population was indeed dispersed in the alveoli of experienced mice (**Figure 9F**). These cells were found to be largely stationary in the resting experienced lungs of PZTD mice, but they exhibited probing behaviors recognizable as cellular extensions and retractions throughout the 30-minute observation period (**Video 1-2**). Sp3 rechallenge did not appear to induce changes in parenchymal B_RM_ cell motility after 24 hours (**Figure 9G** **;** **Video 3-4**). Altogether, the combination of morphometry and live lung imaging reveals that, while B_RM_ cells aggregate around the bronchovascular bundles of Sp-experienced lungs, the alveolar septa also contain a great number of these cells, which diffusely distribute independently of lymphocyte aggregations throughout the parenchyma of Sp-experienced lungs.

## Discussion

Our data show that self-limiting respiratory infections with pneumococcus elicit a heterogeneous and dynamic pool of lung B cells. Re-exposure to pneumococcus 1 week after initial infection stimulates the CD4^+^ cell-dependent accumulation of activated, proliferative EV B cells in a manner that is independent of CD40L and CXCL13. However, the generation of B_RM_ cells require CD40L-signaling during the time of antigen presentation in the lungs. B_RM_ cells can be found abundantly in alveolar tissue outside of peri-bronchovascular leukocyte aggregates, and they do not require CD4^+^ cells for memory recall function.

Full spectrum flow cytometric phenotyping analysis of EV lymphocytes after local exposures to *S. pneumoniae* revealed that B cells clustered into distinct categories based on surface marker profiles. This degree of B cell phenotypic diversity and accumulation was not present in the lung at any timepoint after only 1 exposure to Sp19F. We have shown previously that a singular exposure to Sp19F does not confer heterotypic protection against Sp3 rechallenge, in contrast to IAV immunity models in which longer-term infections and antigen presentation can promote extensive lung remodeling after a single IAV infection (12, 66). Conversely, two exposures to Sp19F administered one week apart generate local heterotypic protection as well as protective PD-L2^+^ B_RM_ cells, which we continue to observe in the lung for up to 6 months after infection (14). Parallel studies examining lung CD4^+^ T cell kinetics in this model reveal a similar pattern in which T cells accumulate rapidly within the first 3 days of the second exposure to Sp19F and contract over the following 4 weeks, yielding a pool of protective T_RM_ cells by 28 dpsi (31, 52). We timed our FTY720, GK1.5, and MR1 administrations to correspond to when these lymphocytes accumulate in the lungs, suggesting that the pulmonary site may be where these lymphocytes are interacting with each other and with antigen. However, because these agents also acted systemically, lymphocyte interactions with each other and with antigen in secondary lymphoid organs would also have been interrupted, and could be important at these times. The role of antigen-specific stimulation of lymphocytes in the lung at this time seems likely, since the second infection was essential, but we cannot differentiate between antigen-specific and bystander lymphocytes until the heterotypic pneumococcal antigens recognized by lung B cells are identified, facilitating development of relevant pneumococcus-specific B cell baits. This also limits the potential for trajectory analysis to extrapolate the path of development from naïve B cells to lung anti-pneumococcal BRM cells (18).

Kinetic analysis of EV B cells showed that several B lymphocyte subsets seed the lung parenchyma and undergo *in situ* cycling in different waves after respiratory pneumococcal exposures. We postulate that the second infection, during which Sp antigen is locally present, is important in promoting the phenotypic changes in the lung necessary to support robust EV B cell populations. Among these populations was a sizeable portion of naïve B cells, likely transiently extravasating into and through the acutely inflamed lung. The significance of these naïve B cells in the infected lung is uncertain. Many of the extravascular B cells in an infected and inflamed lung likely return to the circulation via the lymphatics, underscoring that extravascular location at a given moment does not signify tissue residency.

A parallel has been observed in the formation of lung T_FH_-like cells in response to a mucosal IAV vaccine, which required both a priming exposure to vaccine antigen and reintroduction of antigen within a determined window of time (32). Blockade of lymphocyte recirculation from peripheral lymph nodes during this crucial second local exposure to Sp19F using FTY720 blunted the early establishment of non-naïve and GC B cell populations, as well as the formation of B_RM_ cells. Whether this was due to the hindered influx of B cells from draining lymph nodes or due to the lack of recruited CD4^+^ T cells that can provide help to maturing B cells is unclear and does present a limitation to these studies. In either case, these data showed that the immune cells established initially in the lung with primary Sp19F infection alone were incapable of driving the phenotypic changes in the lung B cell population upon subsequent Sp19F exposure. Although studies to date have not revealed T_FH_-like CD4^+^ cell subsets in pneumococcal-experienced lungs (29, 52), we found that disruption of CD4^+^ cells and CD40L signaling during the period of Sp19F reintroduction abrogated the downstream establishment of lung B_RM_ cells. These findings are in line with studies demonstrating reductions in lung GC and IAV antigen-specific B_RM_ cells after exposure to CD40L blockade during IAV infection (13). We additionally found that other subsets of B cells not traditionally associated with T cell dependence, such as B1 B cells, were also significantly impacted by these interventions. Future studies will focus on elucidating the mechanisms that contribute to the T-dependent genesis of B_RM_ cells and their overall contribution to the lung B cell niche. CD4^+^ cells likely support lung B cells through CD40L-independent means as well, given that the early accumulation of activated CD69^+^ B cells after reinfection was diminished without T cell help, but intact without CD40-CD40L signaling. As was the case for the FTY720 experiments, a significant limitation to these studies is that CD40L blockade is known to have some effects on CD4^+^ T cell populations as well (31, 60). Therefore we are unable to determine whether the observed effects on B cell populations after CD40L blockade were solely due to the treatments or indirectly via impact on CD4^+^ T cells. In addition, it is possible that FTY720, GK1.5, and MR1 could influence immunity against infection and bacterial burdens; if bacterial clearance rates differed among the various treatments that could contribute to the effects observed. Finally, as communicated above, it is not possible to isolate treatments to target lung lymphocytes alone, so we cannot determine whether the interactions that promote lung B cell accumulation take place within or outside the lung is a topic of future study. Our results demonstrate definitive roles for lymphocyte recirculation, CD4^+^ cells, and CD40L-CD40 interaction, but knowledge gaps remain related to what kinds of stimulation are happening in which cells in which tissues.

Chemoattraction to follicles via direct secretion of CXCL13 by T_FH_ cells or indirect promotion of CXCL13 secretion from mesenchymal cells by IL-17^+^ CD4^+^ T cells are known components of T cell help to B cells, including in the lung after viral infection (22, 67). We observed that CXCL13 was dispensable for B_RM_ formation, possibly due to the lack of persistent tertiary lymphoid structures after pneumococcal infections. However, other pro-survival and instructive signals from T cells such as IL-4 and IL-21 contribute to B cell support and could be implicated here (68). This study also raises questions about potential CD4^+^ cell contributions to lung antibody-secreting cells. Studies of plasma cell niches in mucosal tissues have revealed that plasma cell niche requirements vary by location and by Ig isotype (69). The niche requirements for plasma cell survival in the lung are poorly understood, and another important topic for future study.

The exact locations of EV B cells and B_RM_ cells in the lung are not well studied and have predominantly been explored in IAV infection models which generate many long-lasting changes to pulmonary structures including mature iBALT, aberrant keratin 5^+^ epithelial cells, and chronic inflammation (20, 70). In contrast, mice recovered from Sp19F infections exhibit none of these persistent pulmonary abnormalities. Morphometric analysis of lung B cells revealed that B cells occupy both the peri-bronchovascular compartment as well as alveolar regions. By day 35, at least some of these dispersed B cells in alveolar regions were PD-L2^+^ B cells inferred to be B_RM_ cells. These findings corroborate studies using IAV-experienced transgenic reporter mice to track activation-induced cytidine deaminase-activated lung B cells and this combined evidence suggests that B_RM_ cells can indeed be positioned diffusely across alveolar areas. This perhaps increases their area of influence for antigen recognition and facilitates rapid recall responses upon subsequent infection throughout broader lung regions (16). The exact positioning of these cells in the alveolar air space, interstitium, or both is difficult to discern with imaging modalities used here but warrants further study to better understand the molecular cues that lead to the localization of MBCs across alveolar regions and the full extent of their compartmentalized roles in the antigen-experienced lung.

In humans, epidemiologic evidence suggests that frequent asymptomatic exposures to pneumococcus due to the bacteria’s natural reservoir in the nasopharyngeal passages confer serotype-independent immunity against pneumococcal pneumonia (7). Nasopharyngeal colonization in mice also engenders protective immunity against pneumococcal lung infection, including increased plasma cell activity and lung IL-17-secreting T cells (71, 72). The progressive changes in murine lung immune architecture that we observe after multiple exposures to Sp may be extrapolated to humans, which have also been found to harbor B cells with resident and memory phenotypes in histologically unremarkable lungs (14, 26). These studies may inform vaccine research related to pneumococcal pneumonia. The current pneumococcal vaccines use serotype-specific capsular polysaccharides as antigens, which elicit T-independent responses unless conjugated to carrier proteins engineered to elicit T-dependent responses. These vaccines do not induce heterotypic responses or lung-resident immunity, both of which result from recovery from prior pneumonia, and both of which could be useful vaccine improvements. There is precedence for employing mucosal routes of immunization to target mucosal pathogens (73–76). Prior studies aiming to use intranasally-administered inactivated whole cells to elicit serotype-independent immunity have suggested a need for a concomitantly administered adjuvant, as live pneumococci contain elements that induce robust and lasting immune responses compared to inactivated bacteria (12, 77, 78). Future research aims in this field include the identification of safe and effective adjuvants for use in human mucosal vaccine preparations, aiding the development of vaccines that can mobilize the mucosal immune system to confer expanded coverage (73). Overall, our findings suggest that locally administered antigens that trigger T-dependent responses and non-classical GC reactions may be more capable of emulating the natural local serotype-independent immunity found in healthy adults and of preventing pneumonia via lung-localized mucosal immunity.

## Materials and methods

### Mice

All animal protocols used in this study were approved by the Boston University Institutional Animal Care and Use Committee (IACUC). C57BL/6J (B6) mice were purchased from Jackson Laboratory (Bar Harbor, Maine). CD19-Cre+/- PZTD mice were generated by crossing CD19-Cre mice with homozygous PD-L2-ZsGreen-tdTomato-diphtheria toxin knock-in and inducible knockout (PZTD) background as previously described (64); mice were maintained in a specific pathogen-free facility with *ad libitum* food and water. Experiments were initiated when mice were 6-12 weeks of age and included both male and female mice. For i.t. and i.n. instillations, mice were first anesthetized with a mixture of ketamine (50mg/kg) and xylazine (5mg/kg) via i.p. injection prior to instillation. Mice were euthanized by isoflurane overdose. Bilateral thoracotomy was performed to ensure euthanasia before organ collections.

### Bacterial infections

Bacteria were grown on blood agar plates (Trypticase Soy Agar (TSAII) with 5% Sheep Blood, BD Biosciences) for 14-16 hours at 37°C with 5% CO2. Mice were infected with *Streptococcus pneumoniae* serotype 19F (Sp19F EF3030) suspended in sterile saline (1 x 10^6^ bacterial colony forming units (CFUs) in 50 μL) given i.t. into the left lobe. i.t. infections were accomplished by cannulation of the trachea with a 24-gauge angiocatheter (BD 381112) For *Streptococcus pneumoniae* serotype 3 (Sp3 6303, ATCC) challenge infections, 0.5 x 10^6^ CFU suspended in sterile saline were administered to each mouse i.t.

### Tissue collection

Tissues were collected after euthanasia by isoflurane overdose. For BALF collection, the trachea was cannulated with an 18-gauge angiocatheter (BD 381444), and the lungs were lavaged with PBS and centrifuged. Blood was collected from the inferior vena cava into heparin-coated syringes and centrifuged for plasma collection. For lung tissue protein, harvested lungs were homogenized in a bullet blender (Next Advance) in a protease inhibitor solution (Roche 5892791001).

### Lung digestion and flow cytometry

To distinguish IV circulating from EV lung tissue leukocytes, mice anesthetized with ketamine (50mg/kg) and xylazine (5 mg/kg) were injected i.v. with 2 μg of the indicated fluorescent anti-CD45.2 antibody 3 minutes before euthanasia. Single-cell suspensions were prepared by mincing isolated left lung lobes with sterile razors followed by enzymatic digest with type 2 collagenase (Worthington Biochemical LS004176) and DNase I (Sigma-Aldrich DN25) as previously described (12).

All single-cell suspensions were stained with specified fluorescent antibodies (**Supplementary Table 1**) and 7-AAD (BD Biosciences) was used to determine cell viability other than in EdU studies. For EdU studies, the Click-iT AlexaFluor 488 EdU flow cytometry assay kit (Invitrogen C10420) was used per kit instructions, and Zombie UV (BioLegend) dye was used to determine viability. Stained cells were assessed on LSR-II (BD Biosciences) or Cytek Aurora (Cytek Biosciences). All cytometry data were analyzed using FlowJo v10 software (BD Biosciences) and expert gating was used to identify B cell subsets. OMIQ cloud analysis platform (OMIQ, Dotmatics) was used for the computational processing of the data. Briefly, live single EV B cell data exported from FlowJo were concatenated, asinh transformed (cofactor 6000) and selected fluorescence dataset features (B220, IgD, IgM, GL7, CD44, CD62L, CD43, CD11a, CD69, CD73, CXCR5, CD38, PDL2) were clustered via the Phenograph algorithm (kNN = 40, distance metric = Euclidean). The same features were projected into 2D mapping space using opt-SNE dimensionality reduction with principal component analysis-guided map initialization (perplexity = 30) to aid cluster visualization. Phenograph algorithm has identified 22 clusters in the data; 21 clusters with >0.5% frequency were used for downstream analysis.

### Blockade studies

7 days after respiratory infection with Sp19F, mice were treated on days 7, 9, 11, 13, and 15 with various interventions to abrogate processes occurring during repeat exposure to Sp19F administered on day 10, unless otherwise described. The S1P1R inhibitor FTY720 (Sigma SML0700) was delivered i.p. at doses of 1 mg/kg. CD4^+^ cell depletion was achieved using the anti-CD4 monoclonal antibody (clone GK1.5) or IgG2b isotype control (BioXcell) administered i.p. at a dose of 500 μg/100 μL and i.n. at 100 μg/50 μL i.n. CXCL13 was neutralized using the anti-CXCL13 antibody VX5/5378 (graciously provided by Vaccinex) or IgG2a (BioXcell) administered at a dose of 500 μg/100 μL i.p. and 200 μg/50 μL i.n. The CD40L-blocking antibody (clone MR1) or IgG isotype control (BioXcell) was delivered in i.p. instillations (250 μg/100 μL) and i.n. instillations (100 μg/50 μL) per each mouse to prevent CD40L-CD40 interactions. Mice were placed under light sedation with ketamine/xylazine for all i.n. instillations.

### ELISAs

Pneumococcal-specific antibodies were measured in BALF using whole bacterial cell ELISAs. Nunc MaxiSorp plates (Thermo Fisher Scientific) were coated with 1x10^7^ CFU of an acapsular pneumococcal strain derived from a serotype 3 parent. The following HRP-conjugated detection antibodies were used: anti-total IgG (R&D Systems), anti-IgA (Thermo Fisher Scientific), and anti-IgM (Thermo Fisher Scientific). The optical density of each well was determined at 450 nm after the reaction was stopped with 2N H_2_SO_4_.

CXCL12 and CXCL13 protein levels were quantified in homogenized lung tissue using a mouse CXCL12 ELISA kit (R&D Systems MCX120) and a mouse CXCL13 ELISA kit (R&D Systems MCX130) per kit instructions.

### Histology and imaging studies

Slides bearing 5 μm formalin-fixed paraffin-embedded tissue section blocks were deparaffinized in xylene and rehydrated in a graded ethanol series. Slides were exposed to heat-mediated antigen retrieval in a low pH citrate buffer (Vector Laboratories H-3300-250) followed by blocking, primary, and secondary antibody incubation in 5% donkey serum (Abcam ab138579).

For DAB staining, sections were exposed to 3% H_2_O_2_ after antigen retrieval. Slides were treated with the Vectastain ABC-HRP kit (Vector Laboratories PK-4000) followed by development with DAB substrate (Vector Laboratories SK-4100). Sections were counterstained with hematoxylin prior to dehydration and coverslip mounting. Rabbit anti-mouse CD19 antibody (Abcam) was used as the primary antibody and donkey anti-rabbit biotinylated antibody (Jackson Immunoresearch) was used as a secondary antibody. Brightfield images were captured on a Zeiss Axio Scan.Z1 slide scanner with Zeiss ZEN 3.1 Blue software and analyzed using QuPath v0.4.3. Bronchovascular tissue was manually annotated and lumenal areas were excluded using an optimized automated pixel classifier. CD19^+^ cells were enumerated in each tissue compartment using an optimized threshold for automated positive cell detection. Blinded analysis was performed on randomly selected sections.

For IF, rabbit anti-mouse CD19 (Abcam) and rat anti-mouse CD3 (Abcam) were used as primary antibodies and stained with Cy3 donkey anti-rabbit (Jackson ImmunoResearch) and AF647 donkey anti-rat secondary (Jackson ImmunoResearch) antibodies. Sections were treated with TrueVIEW autofluorescence quenching kit (Vector Laboratories SP-8400-15) prior to coverslip mounting with ProLong Gold Antifade mounting media with DAPI (Thermo Fisher P36931). IF images were captured on a Zeiss Axio Observer.Z1 inverted fluorescence microscope with Zeiss ZEN 3.3 Blue software.

Live *ex vivo* imaging of CD19-Cre PZTD mouse lungs was performed as previously described (65). Briefly, extracted mouse lungs were ventilated through a tracheal cannula and perfused through cannulas inserted into the pulmonary artery and the left atrium. A bolus of fluorescent Cascade Blue dextran (Invitrogen) was administered to stain pulmonary vasculature. The lung-heart bloc was placed into crystal ribcage, a synthetic transparent ribcage for microscopy under controlled ventilation and circulation. *Ex vivo* Lungs were imaged using an Olympus FV 3000 inverted laser-scanning confocal microscope and Fluoview acquisition software.

### Statistics

Statistical analyses were performed using GraphPad Prism 9.0. Each graph represents at least two independent experiments. Each individual point on a graph corresponds to an individual animal/sample. Summarized data are shown as means with standard errors. Mann-Whitney tests were used to assess comparisons of two groups, while three or more groups were compared using 2way ANOVA with a Holm-Sidak multiple comparisons test. In multiple comparisons testing, all possible comparisons were made unless otherwise indicated. Two-sided P values were calculated in all cases. Values of p < 0.05 were considered to indicate statistically significant differences.

## Data availability statement

The raw data supporting the conclusions of this article will be made available by the authors, without undue reservation.

## Ethics statement

The animal study was approved by The Institutional Animal Care and Use Committee (IACUC) at Boston University. The study was conducted in accordance with the local legislation and institutional requirements.

## Author contributions

NE: Conceptualization, Funding acquisition, Investigation, Methodology, Formal analysis, Visualization, Writing – original draft. KB: Conceptualization, Investigation, Methodology, Visualization, Formal analysis, Writing – review & editing. ATS: Conceptualization, Funding acquisition, Investigation, Methodology, Formal analysis, Visualization, Writing – review & editing. CL: Investigation, Methodology, Formal analysis, Visualization, Writing - review & editing. EA: Investigation, Methodology, Writing – review & editing. GG: Formal analysis, Investigation, Methodology, Visualization, Writing – review & editing. AM: Methodology, Writing – review & editing. MV: Investigation, Writing – review & editing. RP: Formal analysis, Methodology, Software, Visualization, Writing – review & editing. MB: Investigation, Writing – review & editing. AMS: Methodology, Writing – review & editing. WG: Investigation, Writing – review & editing. CH: Investigation, Writing – review & editing. HB: Investigation, Writing – review & editing. JB: Conceptualization, Writing – review & editing. XV: Resources, Writing – review & editing. KT: Conceptualization, Writing – review & editing. MJ: Conceptualization, Writing – review & editing. LQ: Conceptualization, Writing – review & editing. PM: Conceptualization, Writing – review & editing. HN: Funding acquisition, Resources, Methodology, Writing – review & editing. AB: Formal analysis, Methodology, Data curation, Software, Resources, Writing – review & editing. JM: Conceptualization, Supervision, Funding acquisition, Resources, Writing – review & editing.
