# Supplementary material for: B cells in the pneumococcus-infected lung are heterogeneous and require CD4+ T cell help including CD40L to become resident memory B cells
Source: Front Immunol. 2024 Apr 23;15:1382638. doi: 10.3389/fimmu.2024.1382638 (PMC11074383; doi:10.3389/fimmu.2024.1382638)
Supplement: Supplementary file 5 [file DataSheet_1.pdf]

Supplementary Figure 1

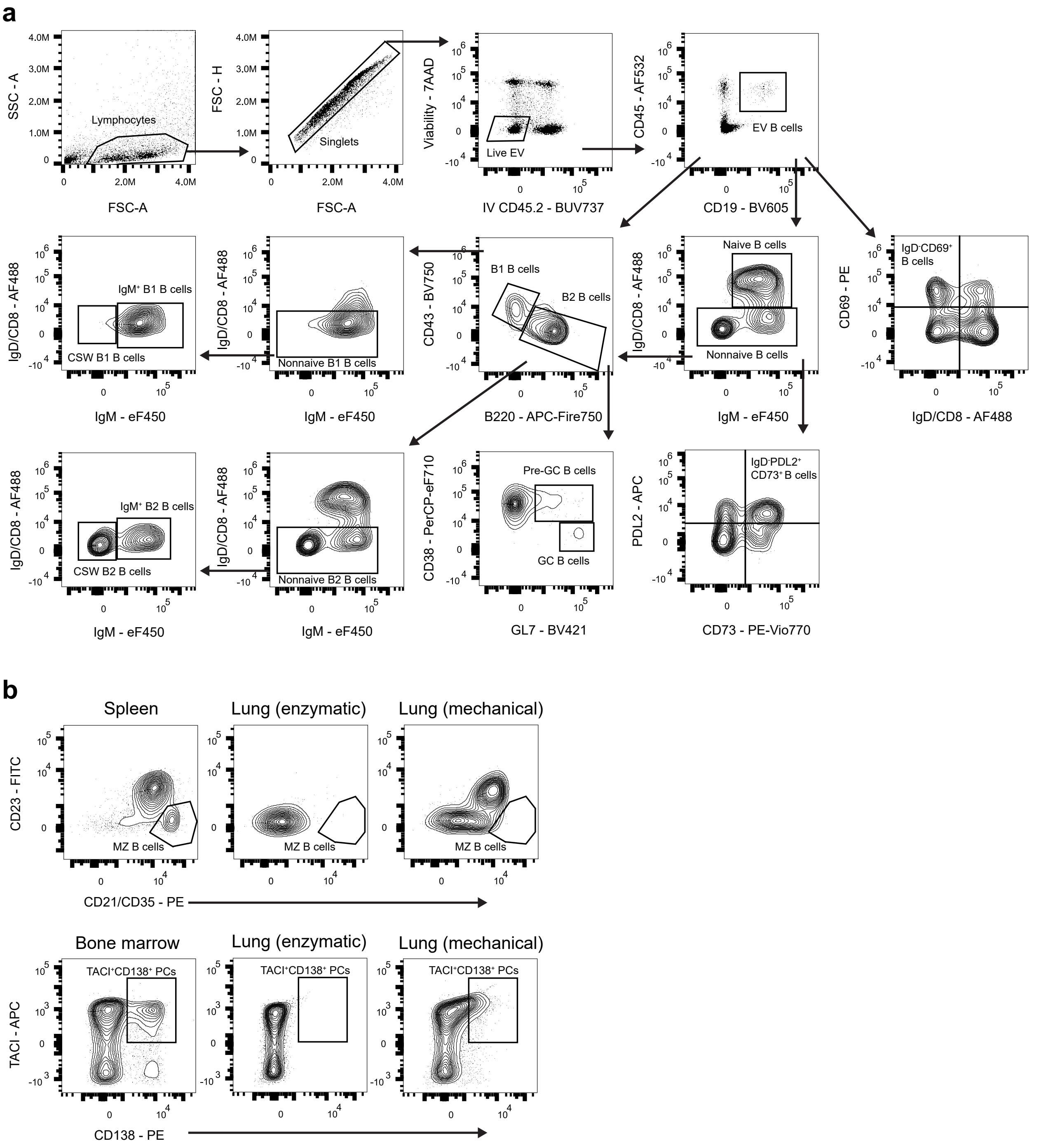

Supplementary Figure 1: Gating strategies to evaluate EV B cell subsets. **a)** Gating strategy used to evaluate live lung EV (i.v. CD45.2^{-}) B cell populations for subset and kinetic studies. **b)** EV marginal zone (MZ) B cells denoted as CD23^{lo}CD21/35^{hi} from single cell suspensions derived from the spleen, lung after collagenase digestion, and lung after mechanical digest (non-enzymatic). EV plasma cells (PCs) denoted as CD138^{+}TACI^{hi} similarly depicted in the bone marrow and lung treated with collagenase digest or mechanical digest.

Supplementary Figure 2

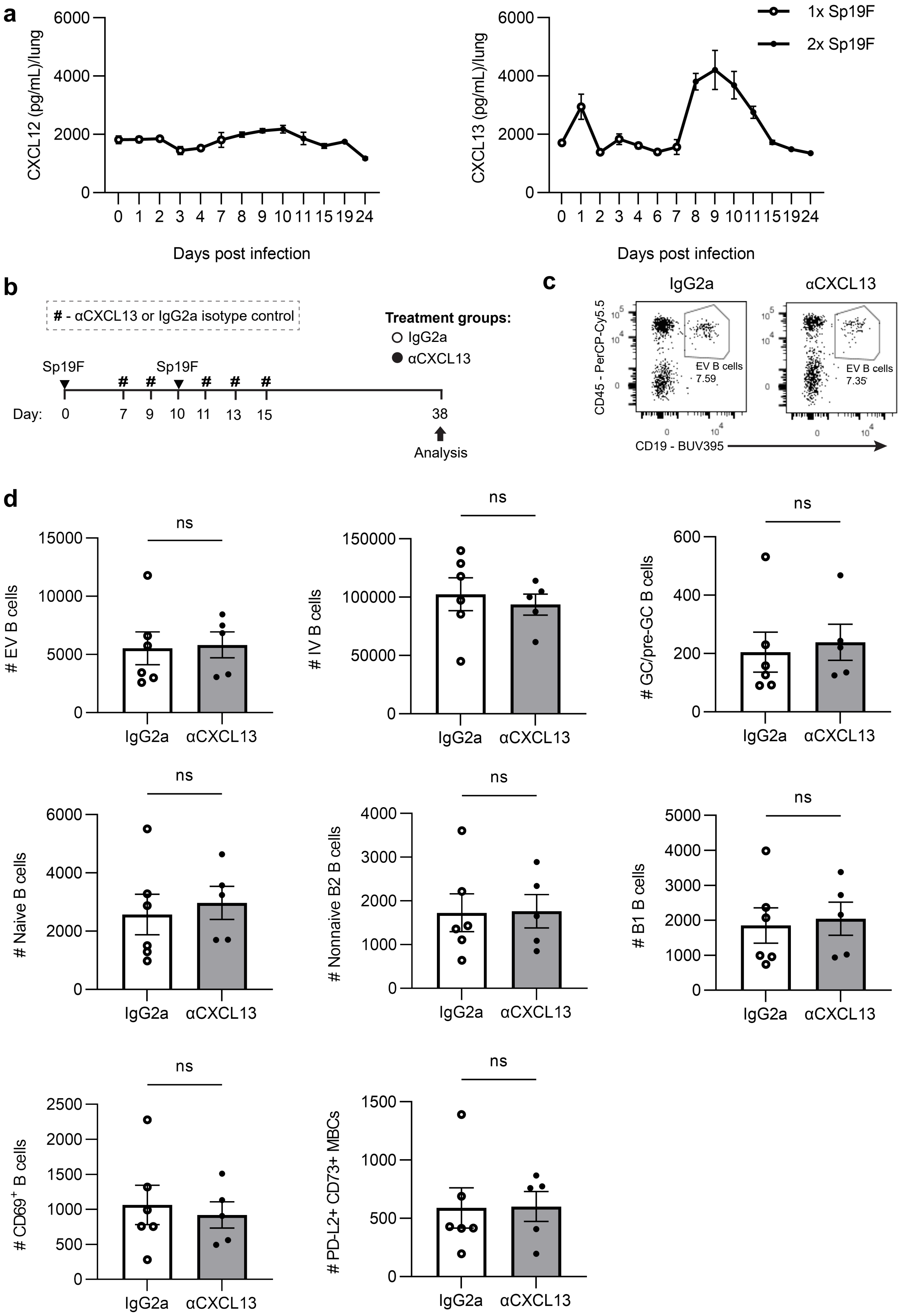

Supplementary Figure 2: CXCL13 increases in the lung after 2 infections but is dispensable for lung EV B cell maintenance. **a)** CXCL12 and CXCL13 protein (pg/mL) in whole lung homogenates collected from mice at the indicated timepoints after 1 and 2 Sp19F infections and detected via ELISAs. **b)** αCXCL13 vs. IgG2a isotype control was administered i.p. and i.n. to B6 mice before, during, and after the second infection with Sp19F as indicated by the # symbols. Mice were euthanized at 28 dpsi for flow cyto-metric analysis. **c)** Representative flow plots depicting EV B cells in Sp-experienced mice treated with αCXCL13 or IgG2a at 28dpsi. **d)** Enumeration of live EV B (i.v.$CD45.2^-CD19^+$), IV B (i.v.$CD45.2^+CD19^+$), GC/pre-GC (i.v.$CD45.2^-CD19^+GL7^+$), naïve B (i.v.$CD45.2^-CD19^+IgD^+$), non-naive B2 B (i.v.$CD45.2^-CD19^+IgD^-CD43^{lo}B220^{hi}$), B1 B (i.v.$CD45.2^-CD19^+CD43^{hi}B220^{lo}$), $CD69^+$ resident (i.v.$CD45.2^-CD19^+CD69^+$) and memory ($IgD^-PD\text{-}L2^+CD73^+$) B cells at 28 dpsi after treatment with αCXCL13 (white bars) or IgG2a (grey bars). n=5-6 per group across 2 independent experiments. Mann-Whitney tests; ns, not significant.

Supplementary Figure 3

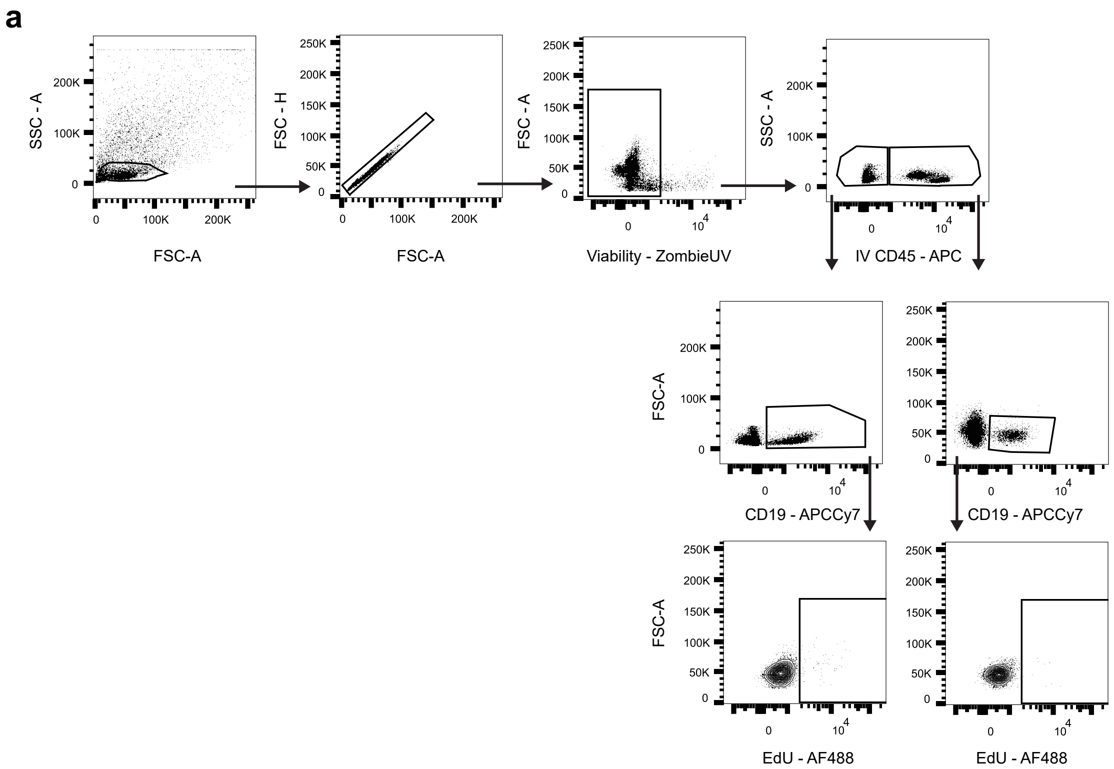

Supplementary Figure 3: Gating strategy to evaluate lung B cell proliferation. **a)** Representative flow plots demonstrating manual gating of EdU^{+} EV B cell subsets based on fluorescence minus one controls.

Supplementary Figure 4

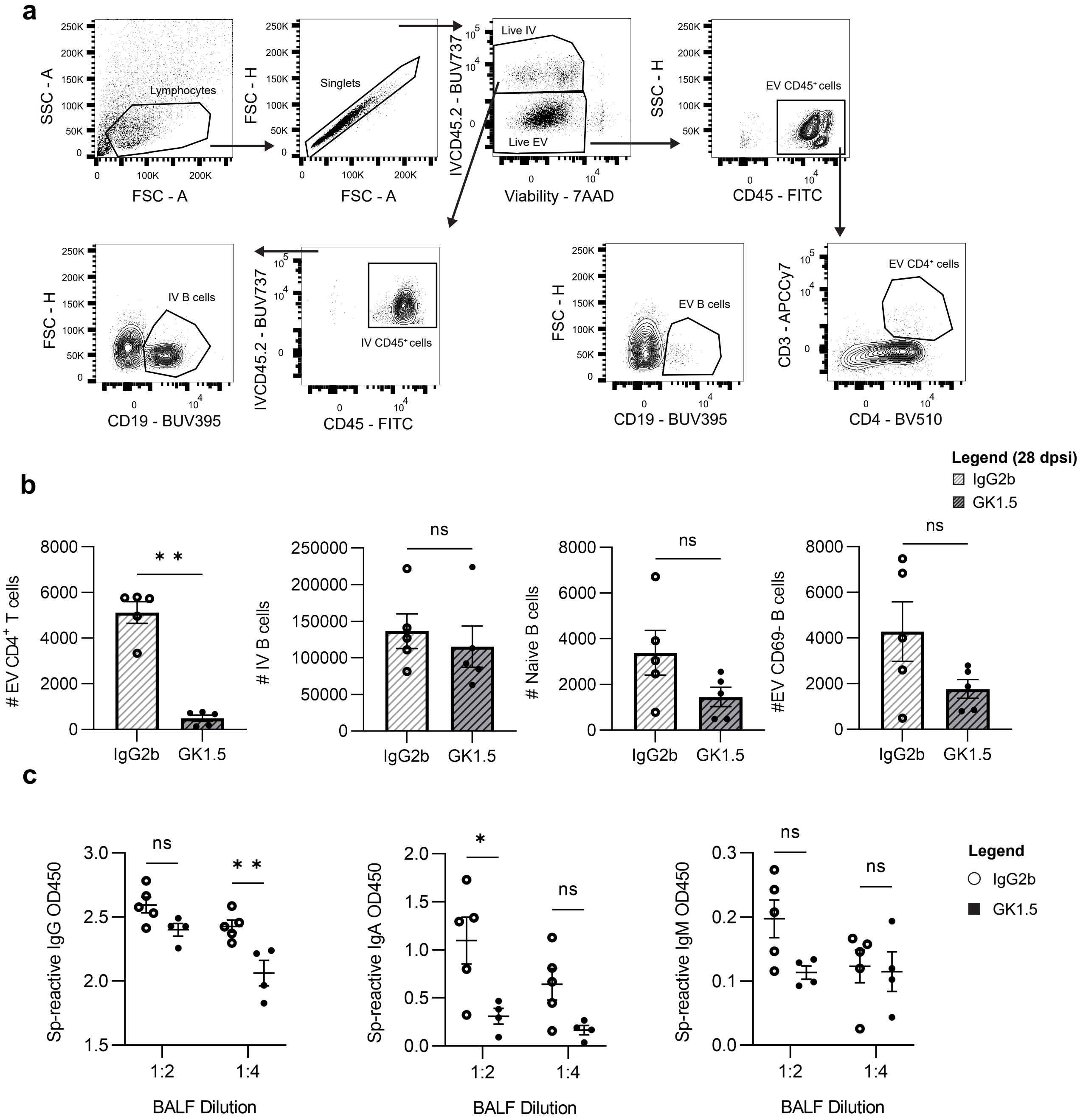

Supplementary Figure 4: Evaluation of lung EV B cells and antibody secreting cell function after $CD4^+$ cell depletion. **a)** Gating strategy to evaluate live IV (i.v. $CD45.2^+$) and EV (i.v. CD45.2-) B cells ($CD45^+CD19^+$) and T cells ($CD45^+CD3^+CD4^+$). **b)** Enumeration of live EV $CD4^+$ T cells, IV B cells, EV naïve B cells (i.v.$CD45.2^-CD19^+IgD^+$), and $CD69^+$ resident (i.v.$CD45.2^-CD19^+CD69^+$) B cells 28 dpsi after treatment with GK1.5 (grey bars with stripes) or IgG2b (white bars with stripes). n = 5 per group across 2 independent experiments. Mann-Whitney tests; **p<0.01; ns, not significant c) Acapsu-lar Sp3-reactive IgG, IgA, and IgM titers in BALF from mice treated with GK1.5 vs. IgG2b were measured via whole-cell pneumococcal ELISAs. n = 4-5 per group across 2 independent experiments. Mann-Whitney tests; *p<0.05; **p<0.01; ns, not significant.

Supplementary Figure 5

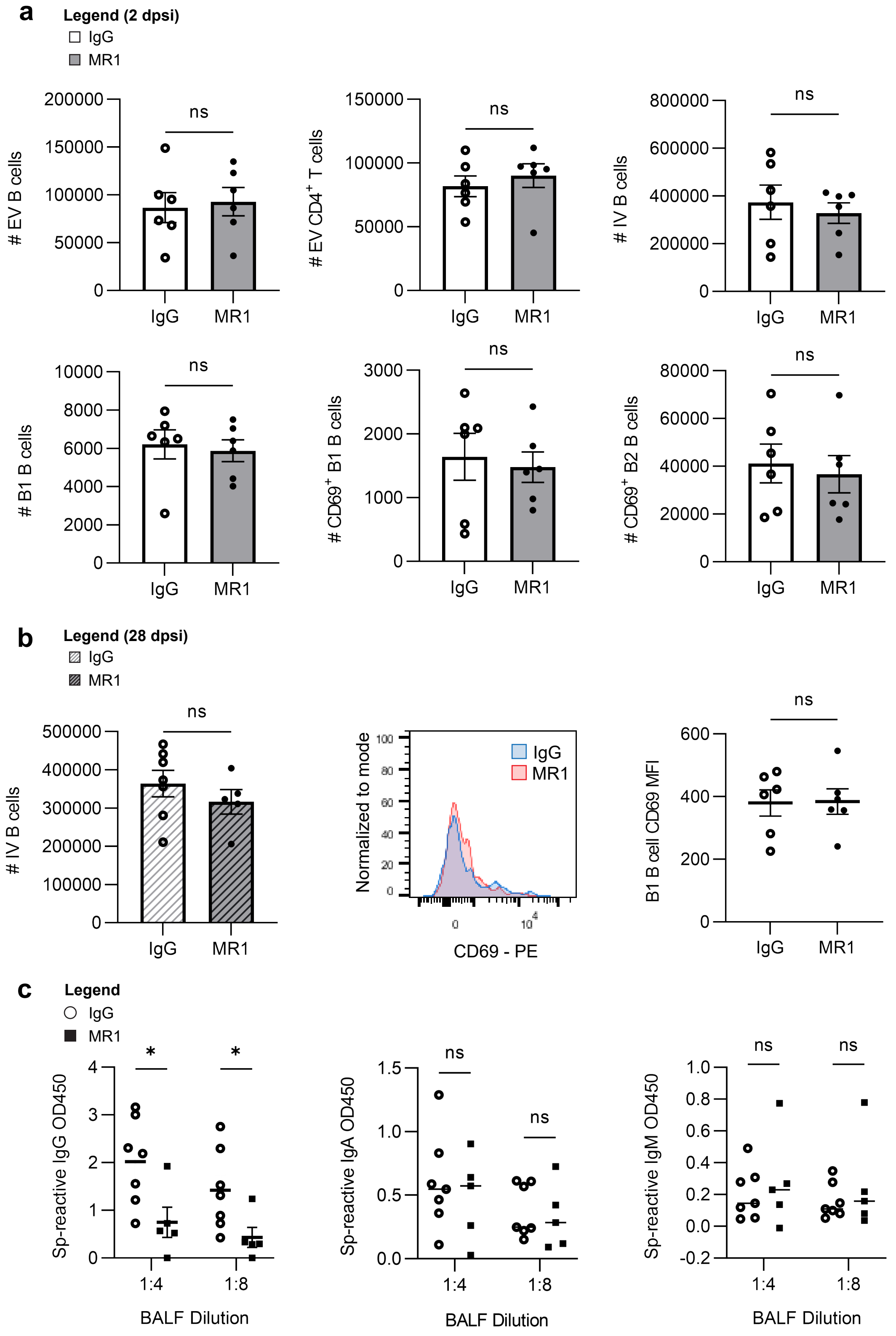

Supplemental Figure 5: Evaluation of lung EV B cells and antibody secreting cell function after CD40L blockade. **a)** Enumeration of live EV B (i.v.CD45.2^{-}CD19^{+}), CD4^{+} T (i.v.CD45.2^{-}CD3^{+}CD4^{+}), IV B (i.v.CD45.2^{+}CD19^{+}), EV B1 B (i.v.CD45.2^{-}CD19^{+}CD43^{hi}B220^{lo}), CD69^{+} B1 B (i.v.CD45.2^{-}CD19^{+}CD43^{hi}B220^{lo}CD69^{+}), and CD69^{+} B2 B (i.v.CD45.2^{-}CD19^{+}CD43^{lo}B220^{hi}CD69^{+}) cells 2 dpsi after treatment with MR1 (grey bars) or IgG (white bars). n=6 per group across 2 independent experiments. Mann-Whitney tests; ns, not significant. b) Enumeration of live IV B (i.v.CD45.2^{+}CD19^{+}) cells and representative overlaid EV B1 B (i.v.CD45.2^{-}CD19^{+}CD43^{hi}B220^{lo}) cell CD69 MFI plots normalized to mode from mice treated with MR1 (red) vs. IgG (blue), with accompanying individual sample MFIs. c) Acapsular Sp3-reactive IgG, IgA, and IgM titers in BALF from mice treated with MR1 vs. IgG2b were measured via whole-cell pneumococcal ELISAs. n = 5-7 per group across 2 independent experiments. Mann-Whitney tests; *$p<0.05$; ns, not significant.

Supplementary Videos

Video 1-2: Live ex vivo imaging of Sp19F-experienced CD19-Cre PZTD mouse lung surface in crystal ribcage62 at the day 35 timepoint with controlled physiologic ventilation and circulation. Timelapse capture of over 30 minutes depicted at 100x speed. TdTomato+ cells (CD19+PDL2+) are observed to exhibit probing behavior among alveoli.

Video 3-4: Live ex vivo imaging of Sp19F-experienced CD19-Cre PZTD mouse lung surface in crystal ribcage62 24 hours after being challenged with Sp3 i.t. with controlled physiologic ventilation and circulation. Timelapse capture over 30 minutes depicted at 100x speed. TdTomato+ cells (CD19+PDL2+) are observed amid alveoli to be largely sessile 24 hpi with Sp3.

Antibodies used for flow cytometry			
**Marker**	**Conjugate**	**Clone**	**Vendor**
B220	APC Fire 750	RA 3-6B2	BioLegend
B220	BUV737	RA3-6B2	BD Biosciences
CD11a	BV786	M17/4	BD Biosciences
CD138	PE	281-2	BioLegend
CD19	BV605	6D5	BioLegend
CD19	BUV395	1D3	BioLegend
CD19	APC-Cy7	6D5	BioLegend
CD19	BUV395	1D3	BioLegend
CD21/35	PE	7.00E+09	BioLegend
CD23	FITC	B3B4	BioLegend
CD25	PECy5.5	PC61	BD Biosciences
CD38	PerCPeF710	90	ThermoFisher
CD3ε	AF647	145-2C11	BioLegend
CD3ε	BV421	145-2C11	BioLegend
CD4	AF700	RM4-4	BioLegend
CD4	BV510	GK1.5	BioLegend
CD43	BV750	S7	BD Biosciences
CD43	PerCPCy5.5	S11	BioLegend
CD44	BV570	IM7	BioLegend
CD45	AF532	30-F11	ThermoFisher
CD45	FITC	30-F11	BioLegend
CD45	PerCPCy5.5	30-F11	BioLegend
CD45	FITC	30-F11	BioLegend
CD45.2	BUV737	104	BD Biosciences
CD62L	BV650	MEL-14	BD Biosciences
CD69	PE	H1.2F3	BioLegend
CD73	PEVio77	REA778	Miltenyi
CD73	APC	TY/11.8	BioLegend
CD8a	AF488	53-6.7	BioLegend
CD8a	BV510	53-6.7	BioLegend
CXCR5	PEeF610	SPRCL5	eBioscience
GL7	Biotin	GL7	BioLegend
IgD	AF488	11-26c	BioLegend
IgD	BV421	11-26c.2a	BioLegend
IgM	eF450	eB121-15F9	ThermoFisher
IgM	PECy7	RMM-1	BioLegend
PD-1	BV510	EH12-287	BioLegend
PD-L2	APC	TY25	BioLegend
PD-L2	PE	TY25	BioLegend

Streptavidin	BV421	n/a	BioLegend
Streptavidin	BV510	n/a	BioLegend
TACI	APC	8F10	BioLegend

Supplementary Table 1: Antibodies used for flow cytometric studies. Cell surface marker, fluorescent conjugate, antibody clone, and vendor information are provided for each antibody.
